# Targeted Perturbation of Mitochondrial Energy Metabolism via Thermoelectric Nanoflowers for Enhanced Cuproptosis Echoed With Immunotherapy

**DOI:** 10.1002/advs.78230

**Published:** 2026-10-14

**Authors:** Haoyang Du, Chao Xu, Hao Wang, Bo Peng, Peng Liu, Wei Gao, Tao Ye, Xinyuan Yu, Ying Shi, Jiaxin Liu, Tiancong Zhao, Bin Sun, Piaoping Yang, Manjie Zhang

**Affiliations:** ^1^ State Key Laboratory of Frigid Zone Cardiovascular Diseases (SKLFZCD), Department of Pharmaceutics, College of Pharmacy Harbin Medical University Harbin People's Republic of China; ^2^ Laboratory of Advanced Materials, Shanghai Key Laboratory of Molecular Catalysis and Innovative Materials, Department of Chemistry Fudan University Shanghai People's Republic of China; ^3^ Key Laboratory of Superlight Materials and Surface Technology, College of Materials Science and Chemical Engineering Ministry of Education, Harbin Engineering University Harbin People's Republic of China

**Keywords:** copper transporter ATPase, cuproptosis, electron transport chain, immunotherapy, thermoelectric materials

## Abstract

Cuproptosis, a novel anti‐cancer mechanism, holds significant potential for triggering sequential immunotherapy to enhance anti‐cancer efficacy. However, this cascade effect is often limited by copper efflux mechanisms mediated by the copper‐transporting ATPase (ATP7B), which reduces intracellular copper accumulation and negates cuproptosis. Here, we designed a mitochondria‐targeting thermoelectric nanoplatform to interfere with the electron transport chain (ETC) of mitochondria, causing adenosine triphosphate (ATP) outage to inhibit ATP7B function, thereby enhancing cuproptosis to achieve a synergistic immunotherapy effect. The nanoplatform was fabricated on a thermoelectric copper telluride (Cu_2‐x_Te) base, doped with bismuth (Bi) to enhance its performance. The morphology was engineered into nanoflowers (NFs) to greatly expand its surface area, achieving a high photothermal conversion efficiency of 66.88%. The established temperature gradient drives a thermoelectric catalytic process on the material's surface, leading to the generation of substantial superoxide anion radicals (•O_2_^−^) to oxidize mitochondrial nicotinamide adenine dinucleotide (NADH), interfering with ETC to impede ATP biosynthesis. We demonstrated that the NFs effectively potentiate cuproptosis and trigger robust immunotherapy, confirming their significant anti‐cancer efficacy both in vitro and in vivo. This study exemplifies a strategy to amplify tumor immunotherapy by enhancing cuproptosis through thermoelectric materials.

## Graphical abstract

We fabricated a thermoelectric nanomedicine to disrupt the ATP energy supply for the copper transporter ATP7B, thereby enhancing cuproptosis and achieving synergistic immunotherapy for anti‐tumor purposes. The fabricated TBCT NFs can efficiently disrupt the electron transport chain function via superoxide‐induced NADH oxidation, leading to ATP depletion. This inhibits ATP7B, enhances copper accumulation, and amplifies cuproptosis. Transcriptome analysis confirmed immune activation, demonstrating a synergistic cuproptosis‐immunotherapy strategy enabled by our thermoelectric TBCT NFs.

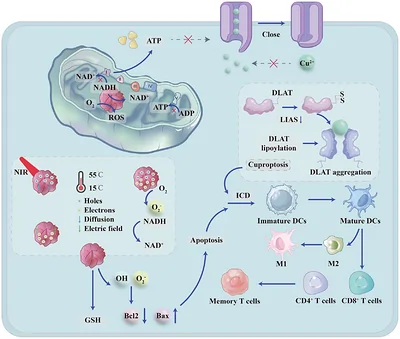

## 1 Introduction

Cuproptosis refers to a novel programmed cell death mechanism mediated by excessive intracellular copper accumulation [1, 2, 3]. It starts with Cu^+^ binding to the lipoylated proteins within the mitochondria, triggering the aggregation of dihydrolipoamide S‐acetyltransferase (DLAT) and the loss of Fe‐S cluster proteins. This cascade induces proteotoxic stress, which further results in cell death [4]. Harnessing cuproptosis for anti‐cancer purposes has attracted great attention as it provides a new programmed cell death mechanism besides the canonical apoptosis mechanism. This direct ion‐induced cell death exhibits high spatiotemporal controllability and precision, rendering it advantageous for tumor therapy. What makes cuproptosis more appealing is its potential to induce immunogenic cell death (ICD) [5, 6, 7]. Specifically, cuproptosis serves as a potent initiator of cell death, thereby liberating damage‐associated molecular patterns (DAMPs) and tumor‐associated antigens that can potentiate immunotherapy. Thus, theoretically, activating cuproptosis could naturally invoke immunotherapy to achieve synergistic anti‐cancer effects.

However, the cuproptosis‐immunotherapy cascade effect hinges on the efficacy of cuproptosis, which requires sufficient copper accumulation within cells [8, 9, 10]. While studies aiming to enhance copper accumulation by designing novel delivery systems have been reported [11], they are mostly categorized as passive strategies. Intrinsically, tumor cells manage to evade cuproptosis by exploiting specialized copper‐transport machinery to maintain cytoplasmic copper homeostasis at low concentrations [12]. During this active cuproptosis‐resistance process, the copper transporter ATPase (ATP7B) is the major transporter used by tumor cells to pump copper ions extracellularly [13]. The confirmation of ATP7B as a copper transporter and its role in maintaining copper homeostasis has been adequately reported [14, 15, 16], and the relevance of ATP7B dysfunction and copper toxicity has been confirmed in pathogenic developments [17, 18]. These studies illustrate the great therapeutic potential of ATP7B‐mediated copper transport.

Currently, translational application studies centered on ATP7B remain largely underexplored. This scarcity is caused by the lack of a clear and efficient solution to inhibit ATP7B function. In this study, we focused on the energy supply required for normal ATP7B function, as this active transport process of copper by ATP7B requires energy derived from ATP hydrolysis [19]. Thus, we proposed that cutting off the ATP7B energy supply is an effective strategy to block copper efflux and subsequently potentiate cuproptosis. More importantly, our core design concept‐cutting off the ATP supply to inhibit ATP7B function‐has not been previously reported as an applied method to realize ATP7B's therapeutic potential, despite its seemingly straightforward nature. This further underscores the scientific contribution of our current work.

ATP is synthesized primarily in mitochondria, the powerhouses of the cell. The core of ATP biosynthesis is oxidative phosphorylation (OXPHOS), a fundamental process in cellular energy metabolism [20, 21]. It involves electron transfer through mitochondrial inner membrane protein complexes (I‐IV) to drive ATP synthesis. Within this system, mitochondrial complex I constitutes the primary site for electron donation to the respiratory chain, catalyzing the oxidation of NADH to NAD^+^ to initiate electron flux through the OXPHOS cascade [22, 23]. Thus, NADH is a vital substrate required by complex I to sustain the OXPHOS system. It has been shown that pharmacological depletion of intracellular NADH inhibits complex I activity, consequently disrupting ATP production [24]. Therefore, depleting NADH is a promising strategy to impede ATP biosynthesis.

Photo‐thermoelectric (PTE) therapy is an emerging modality that leverages temperature gradients to convert thermal energy into electrical fields to interfere with biological processes [25, 26, 27]. In thermoelectric materials, temperature variations induce electron‐hole separation, generating built‐in electric fields [28, 29, 30]. In the cellular environment, such properties can enable thermoelectric materials to initiate redox reactions, generating superoxide anion radicals (•O_2_^−^). These reactions can subsequently oxidize NADH to NAD^+^ via hole‐mediated processes. Thus, the PTE effect could be used to effectively deplete the NADH substrate required by complex I in the ETC, significantly reducing ATP production within cells [31, 32, 33]. Additionally, PTE nanomedicine can also generate cytotoxic reactive oxygen species (ROS) storms that directly eradicate tumor cells, making PTE therapy more effective.

In this study, we fabricated PTE materials possessing high thermoelectric efficiency while simultaneously inducing cuproptosis. Our design originated with copper telluride (Cu_2‐x_Te), renowned for its demonstrated high thermoelectric performance in device applications [34, 35, 36]. To further enhance its thermoelectric properties, we employed a strategic bismuth (Bi) doping approach. This strategy was inspired by reports showing metal doping (notably Bi/In) effectively improves the Seebeck coefficient of thermoelectric materials [37, 38]. We synthesized bismuth‐doped copper telluride (Bi‐Cu_7_Te_4_, BCT) nanoflowers (NFs) using polyacrylic acid (PAA) as a structural template (Scheme 1). These BCT NFs exhibited exceptional thermoelectric properties and were functionalized with triphenylphosphonium (TPP) to confer mitochondria‐targeting capability. Functionally, the resulting TPP‐Bi‐Cu_7_Te_4_ NFs (TBCT NFs) precisely accumulate within mitochondria to release copper ions and, crucially, deplete the NADH substrate via the thermoelectric effect, thereby suppressing ATP synthesis. We demonstrated that such energy depletion can effectively attenuate the copper transport function of ATP7B, inhibiting copper efflux and potentiating cuproptosis. This enhanced cuproptosis induced ICD, promoting dendritic cell (DC) maturation and macrophage polarization towards the M1 phenotype. This immune remodeling enhanced cytotoxic T cell differentiation and established long‐term immunological memory. Thus, our thermoelectric NFs can achieve efficient tumor immunotherapy through amplified cuproptosis, and we demonstrated the synergistic anti‐cancer effect both in vitro and in vivo.

**SCHEME 1:**
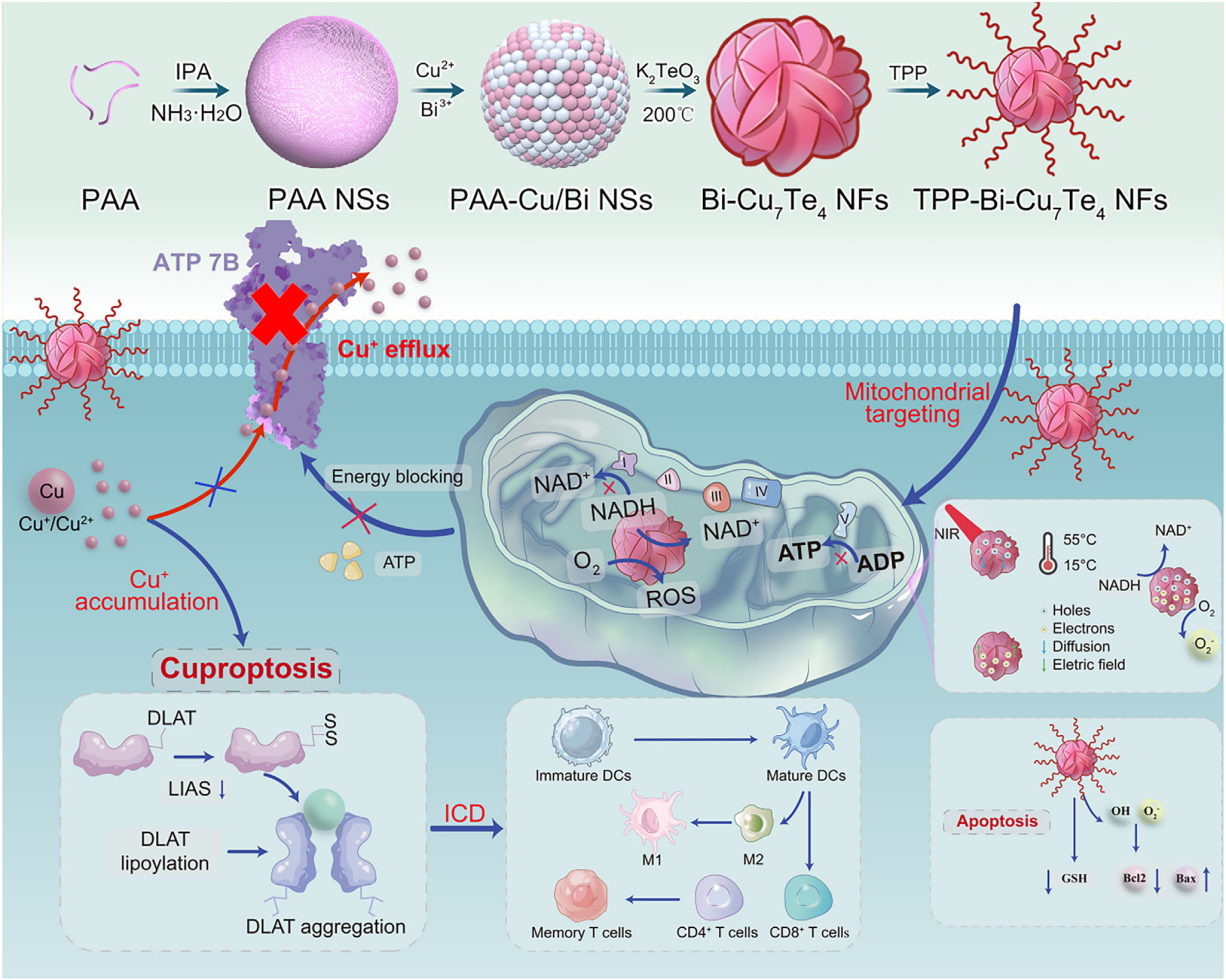
Design and fabrication of mitochondria‐targeting thermoelectric TBCT NFs capable of impeding ATP biosynthesis to potentiate cuproptosis and to trigger ICD for enhanced anti‐cancer therapy.

## 2 Results and Discussion

### 2.1 Synthesis and Characterization of TPP‐Bi‐Cu_7_Te_4_ Nanoflowers (TBCT NFs)

The synthetic route for TBCT NFs is schematically illustrated in Figure 1A. Initially, PAA self‐assembled into nanospheres (NSs) in a water/isopropanol (1:6 v/v) mixed solvent. These PAA NSs then chelated Cu^2+^ and Bi^3+^ via carboxylate‐metal coordination, forming PAA‐Cu/Bi NSs. Subsequent hydrothermal method with potassium tellurite (K_2_TeO_3_) at 200°C for 12 h yields BCT NFs. Finally, surface conjugation with TPP‐NH_2_ through EDC/NHS‐mediated condensation reaction produces mitochondria‐targeted TBCT NFs. Transmission electron microscopy (TEM) images (Figure 1B,C) confirm the successful synthesis of PAA‐Cu/Bi NSs (diameter of 150 ± 5 nm) and TBCT NFs (diameter of 200 ± 8 nm). High‐resolution transmission electron microscopy (HRTEM) analysis of the TBCT NFs intermetallic revealed an interfacial lattice spacing of 0.208 nm, which was assigned to the (110) crystal planes (Figure 1D). Further morphological characterization by scanning electron microscopy (SEM) (Figure 1E) reveals that TBCT NFs exhibit hierarchical NFs architectures assembled from interlaced nanosheets. The material's distinct hierarchical structure creates abundant interfaces for phonon scattering, which reduces lattice thermal conductivity and ultimately boosts thermoelectric efficiency [39]. Elemental mapping analysis (Figure 1F) demonstrates the uniform dispersion of Cu, Bi, and Te throughout the NFs, while the detected P signal verifies successful TPP surface conjugation. Furthermore, Fourier‐transform infrared spectroscopy (FTIR) corroborated successful TPP conjugation on TBCT NFs surfaces (Figure 1G). The energy‐dispersive X‐ray spectroscopy (EDS) analysis further confirmed the co‐presence of Cu, Bi, and Te elements. (Figure 1H). Zeta potential measurements (Figure S1) demonstrated a significant potential reversal from ‐20.7 ± 1.2 mV for BCT NFs to +28.3 ± 0.9 mV for TBCT NFs (1 mM PBS, pH 7.4), reflecting the successful introduction of cationic triphenyl phosphonium groups of TPP. X‐ray diffraction (XRD) patterns (Figure 1I) demonstrate well‐indexed peaks matching the standard Cu_7_Te_4_ phase (JCPDS 45–1287). X‐ray photoelectron spectroscopy (XPS) analysis was employed to probe the elemental oxidation states in BCT NFs. The survey scan (Figure 1J) clearly identifies characteristic photoelectron peaks corresponding to Cu 2p, Bi 4f and Te 3d core levels. Cu 2p XPS spectra (Figure 1K) resolve doublet peaks at binding energies of 932.3 eV (2p_3/2_) and 952.1 eV (2p_1/2_) with a spin‐orbit splitting of 19.75 eV. Deconvolution analysis indicates a mixed‐valence state composed of 79.7% Cu^+^ (centered at 932.3 eV) and 20.3% Cu^2+^ (934.5 eV). While the major state Cu^+^ serves as a driver for cuproptosis by inducing DLAT oligomerization, the minor Cu^2+^ state is a substrate for Fenton‐like reaction to generate hydroxyl radicals (•OH) to exert CDT [40]. The Bi 4f spectrum (Figure 1L) exhibit characteristic doublets at 158.75 and 164.05 eV, while Te 3d spectra (Figure 1M) show peaks at 575.4 and 585.85 eV, and carbon element is in the 1s reference spectrum (Figure S2). Dynamic light scattering (DLS) measurements revealed that TBCT NFs exhibit a hydrodynamic diameter of 250 ± 3 nm with narrow size distribution (polydispersity index, PDI = 0.207 ± 0.014) in phosphate‐buffered saline (Figure 1N). The TBCT NFs were stable within in the seven days incubation in RPMI 1640 (10% FBS), and in other diverse media (Figure S3a,b). The rate of Cu released from TBCT NFs under varying conditions was quantified using inductively coupled plasma optical emission spectrometry (ICP‐OES). The material exhibited pH‐dependent release profiles (Figure S4): 40.5% and 32.5% of Cu was liberated at mildly acidic pH 5.5 and 6.5, respectively, whereas only 9.1% was released at physiological pH 7.4, indicating distinct pH‐responsive behavior.

**FIGURE 1:**
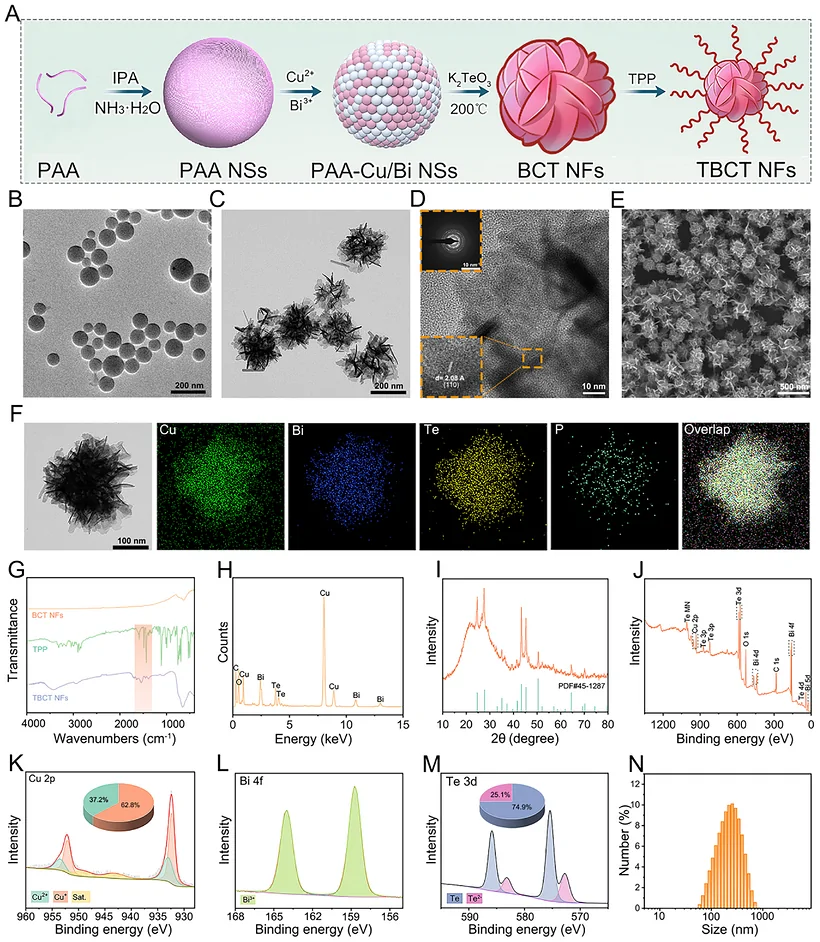
Preparation and characterizations of TBCT NFs. (A) Schematic illustration of the synthetic procedure of TBCT NFs. TEM images of (B) PAA‐Cu/Bi NSs and (C) TBCT NFs. (D) HRTEM image of TBCT NFs. (E) SEM image of TBCT NFs. (F) The elemental mapping images of TBCT NFs. (G) FTIR spectra of BCT NFs, TPP, and TBCT NFs. (H) EDS survey spectrum. (I) XRD patterns. (J) Survey XPS spectrum of BCT NFs. (K) Cu 2p, (L) Bi 4f, and (M) Te 3d core‐level XPS spectra. (N) DLS analysis of TBCT NFs.

### 2.2 Photothermal and Thermoelectric Performance of TBCT NFs

We first characterized the photothermal effect of TBCT NFs, since temperature gradient generation is a prerequisite for thermoelectric conversion. The optical properties of TBCT NFs were quantified using UV‐vis‐NIR spectroscopy (Figure 2A). Concentration‐dependent absorbance enhancement at 808 nm yielded a mass extinction coefficient of 8.32 L·g^−1^·cm^−1^, with linear correlation (R^2^> 0.99) confirmed in Figure 2B. Under 808 nm irradiation, progressive temperature elevation was observed with increasing concentration (Figure 2C). Notably, the 50 µg mL^−1^ dispersion exhibited rapid photothermal response, achieving a temperature increase of 44.3°C in 5 min, which significantly surpassed the minimal thermal fluctuation observed in water. Furthermore, TBCT NFs dispersion temperature exhibited a power‐dependent elevation profile under 808 nm irradiation (Figure 2D), where increased laser power density caused larger temperature elevation. Cyclic heating‐cooling measurements over three consecutive laser on/off cycles demonstrated exceptional photothermal cycling stability (Figure 2F). Concurrently, the photothermal conversion efficiency (*η*) for TBCT NFs was calculated as 66.88% (Figure 2E).

**FIGURE 2:**
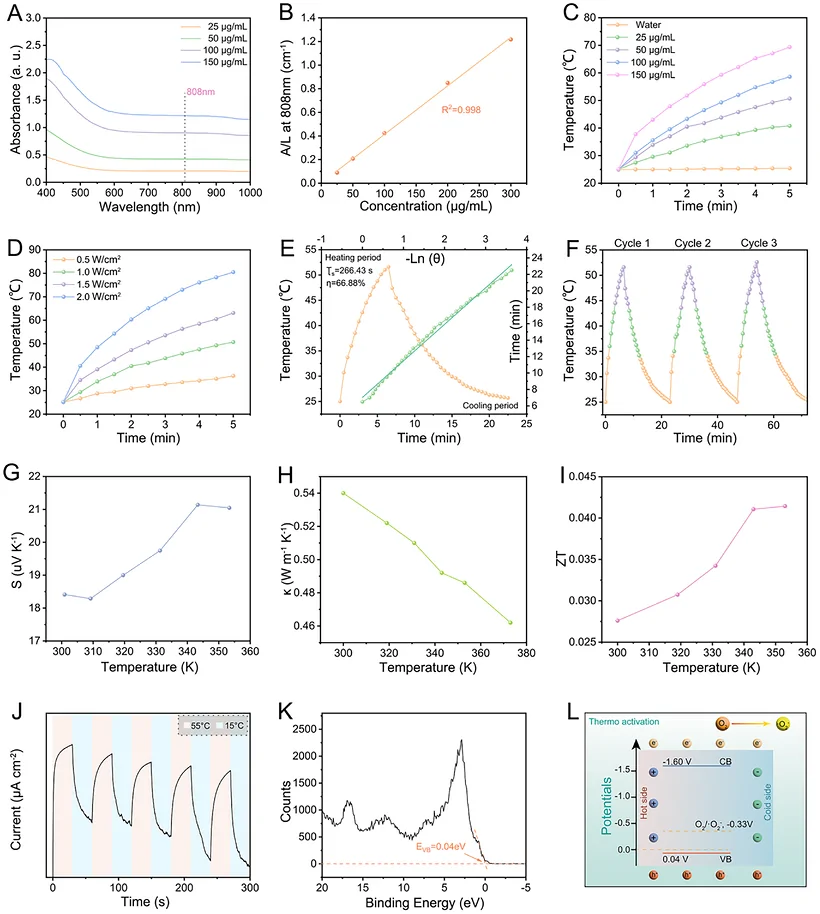
Photothermal evaluation and thermoelectric mechanism of TBCT NFs. (A) UV‐vis absorbance spectra of the TBCT NFs at varying concentrations. (B) Fitted mass extinction coefficient of TBCT NFs under 808 nm irradiation. (C) Photothermal heating curves of TBCT NFs under 808 nm irradiation (1 W cm^−2^) at different concentrations for 5 min. (D) Photothermal heating curves of TBCT NFs (50 µg mL^−1^) upon 808 nm irradiation. Different power densities of laser were considered. E) Photothermal profile of TBCT NFs (50 µg mL^−1^) under 808 nm irradiation (1 W cm^−2^) and the subsequent natural cooling phase. The green line represents the linear fitting of the cooling data, plotted as −lnθ versus time. (F) Cycling performance of TBCT NFs under repeated photothermal heating and natural cooling. (G) Seebeck coefficient, (H) thermal conductivity κ, and (I) dimensionless figure of merit ZT for TBCT NFs. (J) Thermoelectric response of TBCT NFs to periodic temperature changes. (K) Valence band XPS spectrum of TBCT NFs, representing the valence band maximum position. (L) Schematic illustration of the energy bands of TBCT NFs, showing that the formation of •O_2_^−^ free radical is possible.

The thermoelectric performance of TBCT NFs was further evaluated using the figure of merit (ZT), a well‐established dimensionless metric to characterized the efficiency of thermoelectric materials that has a definition as follows [24]:

$$\mathrm{ZT}=S^{2}\sigma T/\kappa$$

where S represents the Seebeck coefficient, σ and κ denote the electrical and conductivity of the material, respectively, and T is the absolute temperature. A higher ZT value means better thermoelectric conversion efficiency. We first measured the electrical conductivity (σ) of the TBCT NFs via spark plasma sintering (SPS). Within the temperature range from room temperature (300 K) to 353 K, the TBCT NFs exhibit excellent electron transport (Figure S5). At room temperature, the electrical resistivity (ρ) of TBCT NFs was measured as low as 0.68 mΩ·cm, corresponding to a high electrical conductivity (σ = 1/ρ) of 146.5 × 10^3^ S m^−1^. Clearly, ρ increased slowly from 300 to 353 K, followed by a more rapid increase, reaching 0.77 mΩ·cm at 353 K, which corresponds to a calculated σ of 128.8 × 10^3^ S m^−1^. The decreasing σ of TBCT NFs with increasing temperature indicates transport behavior characteristic of a highly degenerate semiconductor.

The Seebeck coefficient (S) is a fundamental parameter that defines a thermoelectric material's capacity to generate an electrical voltage from a temperature difference. It is mathematically represented by the equation S = −ΔV/ΔT. The S of TBCT NFs was measured from 298 to 353 K, where S increased from 18.4 µV K^−1^ to 21 µV K^−1^ (Figure 2G). The positive value of S signifies p‐type semiconductor behavior in TBCT NFs. Based on these S and σ values, the power factor (PF) was determined to be 49.6 µW m^−1^ K^−1^ at room temperature and 57 µW m^−1^ K^−1^ at 353 K.

Furthermore, the thermal conductivity (κ) of the TBCT NFs was measured by a range of temperatures. As shown in Figure 2H, the κ values decreased with increasing temperature, remaining below 0.6 W m^−1^ K^−1^ in all cases. A minimum value of 0.46 W m^−1^ K^−1^ was recorded at 373 K. Compared to other existing thermoelectric materials, this represents an exceptionally low thermal conductivity within the lower temperature range, which is highly advantageous for thermoelectric applications (Figure S6) [41, 42, 43]. The corresponding ZT values are plotted in Figure 2I, demonstrating that the combination of a moderate S and an ultralow κ contributed to a value of 0.027 at room temperature, which increased to 0.041 at 353 K. These results demonstrate the thermoelectric properties of TBCT NFs, which establish a foundation for subsequent experiments.

Electrochemical cycling under thermal perturbation (55°C/15°C) demonstrated stabilized current responses over five heating/cooling cycles (Figure 2J), confirming enhanced charge carrier separation and mobility‐a critical attribute of efficient thermoelectric nanomaterials. Valence band XPS analysis (Figure 2K) determined the valence band maximum (VB) at 0.04 eV. UV‐vis diffuse reflectance spectroscopy (DRS) revealed a direct bandgap (E_g_) of 1.64 eV (Figure S7). The conduction band minimum (CB) was derived as E_CB_ = E_VB_—E_g_, yielding ‐1.60 eV versus vacuum. As schematized in Figure 2L, this CB energetically exceeds the O_2_/·O_2_^−^ redox potential (‐0.33 V vs. NHE) by ‐1.06 eV, demonstrating that the electric effect of the TCBT NFs induced by temperature gradient can enable electron transfer for •O_2_^−^ generation.

### 2.3 Photothermoelectric Effect and NADH Depletion Ability of TBCT NFs

After characterizing the excellent thermoelectric property of the TBCT NFs, we next evaluate the efficiency of TBCT NFs in depletion NADH, as depleting NADH is our designing principle to interfere with ATP biosynthesis. We summarized the mechanism in Figure 3A which reads that, under laser irradiation, a temperature gradient will be generated across the TBCT NF surface that drives electron‐hole separation via the thermoelectric effect, which further triggers the oxidation of NADH to NAD^+^ and the reduction of O_2_ to •O_2_^−^. We thus first measured the generation of •O_2_^−^ by TBCT NFs under laser irradiation followed by ice incubation, and to assess the thermoelectric performance, we applied three consecutive NIR laser on/off cycles each comprising 2 min 808 nm irradiation, followed by ice‐pack incubation (1 min)Thisis treatment is termed as TBCT NFs+NIR+ICE group. The generation of ·O_2_^−^ by the thermoelectric effect was monitored using dihydrorhodamine 123 (DHR123). The fluorescence intensity of DHR123 progressively increased with the number of photothermal cycles applied to the TBCT NFs (TBCT NFs+NIR+ICE) (Figure 3B), demonstrating that the thermoelectric effect facilitates •O_2_^−^ production. Furthermore, electron spin resonance (ESR) spectroscopy employing 5,5‐dimethyl‐1‐pyrroline N‐oxide (DMPO) as a spin trap confirmed the presence of •O_2_^−^. As shown in Figure 3C, the characteristic signal observed during the thermal cycles matched that of the DMPO/•O_2_^−^ adduct, providing direct evidence for •O_2_^−^ generation.

**FIGURE 3:**
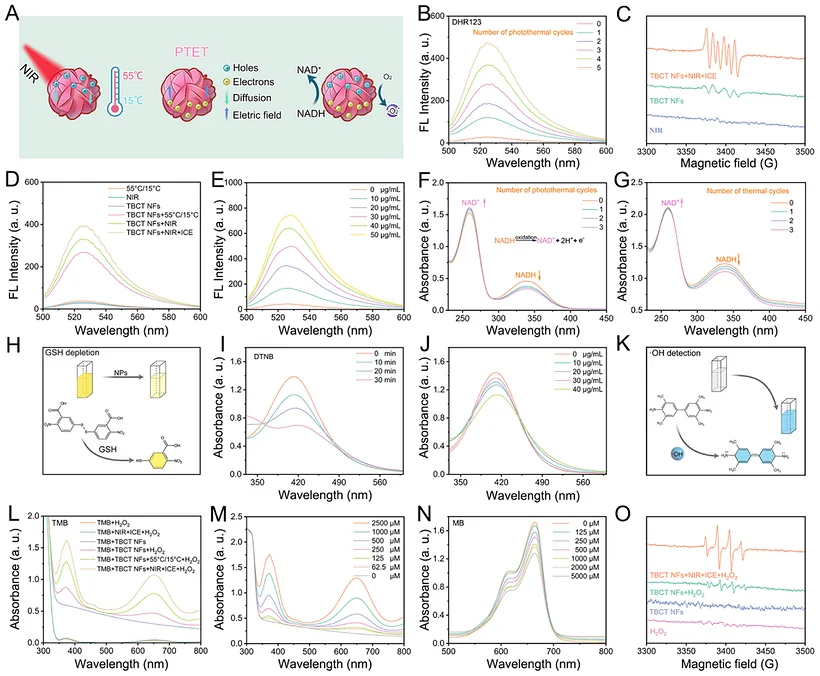
PTET and chemodynamic evaluation. (A) Schematic illustration of the PTET process. (B) Fluorescence spectra of DHR123 for five cycles of 808 nm irradiation (1.0 W/cm^2^). (C) ESR signals for •O_2_^−^ detection across various experimental groups. (D, E) DHR123 recorded under various conditions and at different TBCT NFs concentrations, respectively. (F) NADH oxidation by TBCT NFs during three cycles of laser irradiation. (G) NADH oxidation during thermal cycles. (H) Schematic of the GSH assay. (I) UV‐vis absorption spectra of 5,5’‐dithiobis‐(2‐nitrobenzoic acid) (DTNB) for different time and (J) different concentration. (K) Schematic diagram of TMB testing for •OH. (L, M) UV‐vis absorption spectra of oxidized TMB recorded under various conditions and at different H_2_O_2_ concentrations, respectively. (N) UV‐vis absorption spectra of oxidized TMB recorded at different H_2_O_2_ concentrations. (O) Detection of •OH by ESR spectroscopy using DMPO as the spin trap across different experimental groups.

The generation of •O_2_^−^ under different temperature gradients were also investigated. We consider a fixed temperate gradient (55°C/15°C), a laser irradiation elevated temperature followed by nature cooling (NIR treatment), and a laser irradiation elevated temperate followed ice‐accelerated cooling (NIR+ICE treatment) (Figure 3D). Fluorescence intensity analysis revealed relatively weak signals in both the TBCT NFs group and the 55°C/15°C group. In contrast, significantly enhanced fluorescence was observed in the TBCT NFs+NIR+ICE group, the TBCT NFs+55°C/15°C (without laser), and TBCT NFs+NIR group. Critically, the fluorescence intensity in the the TBCT NFs+NIR+ICE group substantially exceeded that observed in either the TBCT NFs+55°C/15°C group or the TBCT NFs+NIR group alone. These findings strongly support the correlation between •O_2_^−^ generation and the thermoelectric effect. Furthermore, the concentration‐dependent generation of •O_2_^−^ during laser‐induced thermal cycling (TBCT NFs+NIR+ICE) was assessed using DHR123 across varying concentrations of TBCT NFs (Figure 3E), which shows that the ability of •O_2_^−^ is positively correlated with the concentration of the TBCT NFs suspensions.

NADH and NAD^+^ serve as critical cofactors in cellular OXPHOS. The design principle of TBCT NFs leverages their thermoelectric properties to convert NADH to NAD^+^, thereby inhibiting the OXPHOS process by depleting the NADH supply. The interconversion between NADH and NAD^+^ was monitored by UV‐vis absorption spectroscopy. As shown in Figure 3F, increasing the number of laser‐induced thermal cycles applied to the TBCT NFs resulted in a progressive decrease in the characteristic absorption peak of NADH and a concomitant increase in the peak corresponding to NAD^+^. These spectral changes demonstrate that TBCT NFs effectively oxidize NADH under laser‐induced thermal cycling (TBCT NFs+NIR+ICE) conditions. To further confirm the NADH‐oxidizing capability of TBCT NFs under thermally varying conditions independent of laser irradiation, controlled heating/cooling cycles were implemented using alternating water baths (55°C/15°C) (Figure 3G). The observed NADH oxidation in this system verifies that TBCT NFs retain this functionality under temperature gradients alone. Collectively, these results substantiate that the thermoelectric effect is the underlying mechanism driving NADH conversion by TBCT NFs.

Glutathione (GSH) plays a critical role in cellular redox homeostasis by mitigating ROS‐induced damage. The effect of TBCT NFs on GSH depletion was quantified using UV‐vis spectrophotometry (Figure 3H). Analysis revealed a time‐dependent decrease in GSH levels following exposure to TBCT NFs (Figure 3I). Furthermore, GSH content exhibited a concentration‐dependent reduction in response to increasing concentrations of TBCT NFs (Figure 3J). Collectively, these results demonstrate that TBCT NFs effectively deplete GSH and disrupt intracellular redox homeostasis within tumor cells.

Furthermore, TBCT NFs exhibit exceptional chemodynamic performance, enabling highly efficient generation of •OH. To detect •OH production, 3,3′,5,5′‐tetramethylbenzidine (TMB) was employed as an indicator, since •OH can oxidize TMB to produce blue oxTMB (Figure 3K). UV‐vis absorption spectroscopy revealed significant absorption peaks at 370 and 652 nm for the TBCT NFs +H_2_O_2_ system, indicating ·OH generation via a Fenton‐like reaction (Figure 3L). Moreover, through a mixed‐valence Cu^+^/Cu^2+^ strategy, TBCT NFs enhance the efficiency of the Fenton‐like reaction, enabling effective •OH generation even at H_2_O_2_ concentrations as low as 500 µM (Figure 3M). Concentration‐dependent experiments further confirmed that •OH production exhibits a linear positive correlation with the concentration of TBCT NFs (Figure S8a). Additionally, •OH yield increased with prolonged reaction time between TBCT NFs and H_2_O_2_ (Figure S8b). The generation of •OH across varying H_2_O_2_ concentrations was further validated using methylene blue (MB) degradation assays (Figure 3N), with comprehensive evaluations under different conditions, concentrations, and time points provided in Figure S9. ESR spectroscopy with DMPO spin trapping confirmed ·OH formation (Figure 3O), showing a substantial enhancement in signal intensity under laser‐induced thermal cycling. Collectively, these results demonstrate that TBCT NFs possess superior Fenton‐like activity for efficient •OH generation in the presence of H_2_O_2_, while simultaneously producing •O_2_^−^ via the thermoelectric effect, thereby synergistically promoting both NADH oxidation and •OH production.

### 2.4 ATP7B Function Inhibition by TBCT NFs and the Resultant Enhanced Cuproptosis

To investigate the impact of thermoelectric effects on cuproptosis, 808 nm irradiation was employed as the heat source combined with ice‐pack cooling to establish a well‐defined thermal gradient (Figure 4A). Using triple‐negative breast cancer cells (4T1) as an in vitro model, we then evaluated the ability of TBCT NFs to induce cuproptosis and their antitumor efficacy. First, a Cell Counting Kit‐8 (CCK‐8) assay was performed to assess the therapeutic potential of TBCT NFs under different conditions. As illustrated in Figure 4B, TBCT NFs (20 µg mL^−1^) induced significant cytotoxicity, with a sharp reduction in cell viability after 12 h of NIR irradiation or combined NIR and ice‐pack cooling (NIR+ICE) conditions. This synergistic cytotoxicity stemmed from the combined photothermal and thermoelectric effects, which collectively induced hyperthermia, generated ROS, and enhanced cuproptosis. Moreover, to assess the biocompatibility of TBCT NFs, we examined their cytotoxicity against L929 cells at 12 h. The results showed only negligible cytotoxic effects at concentrations as high as 50 µg mL^−1^ (Figure 4C). These results demonstrate the remarkable antitumor efficacy of TBCT NFs and provide a foundation for further experimental investigations.

**FIGURE 4:**
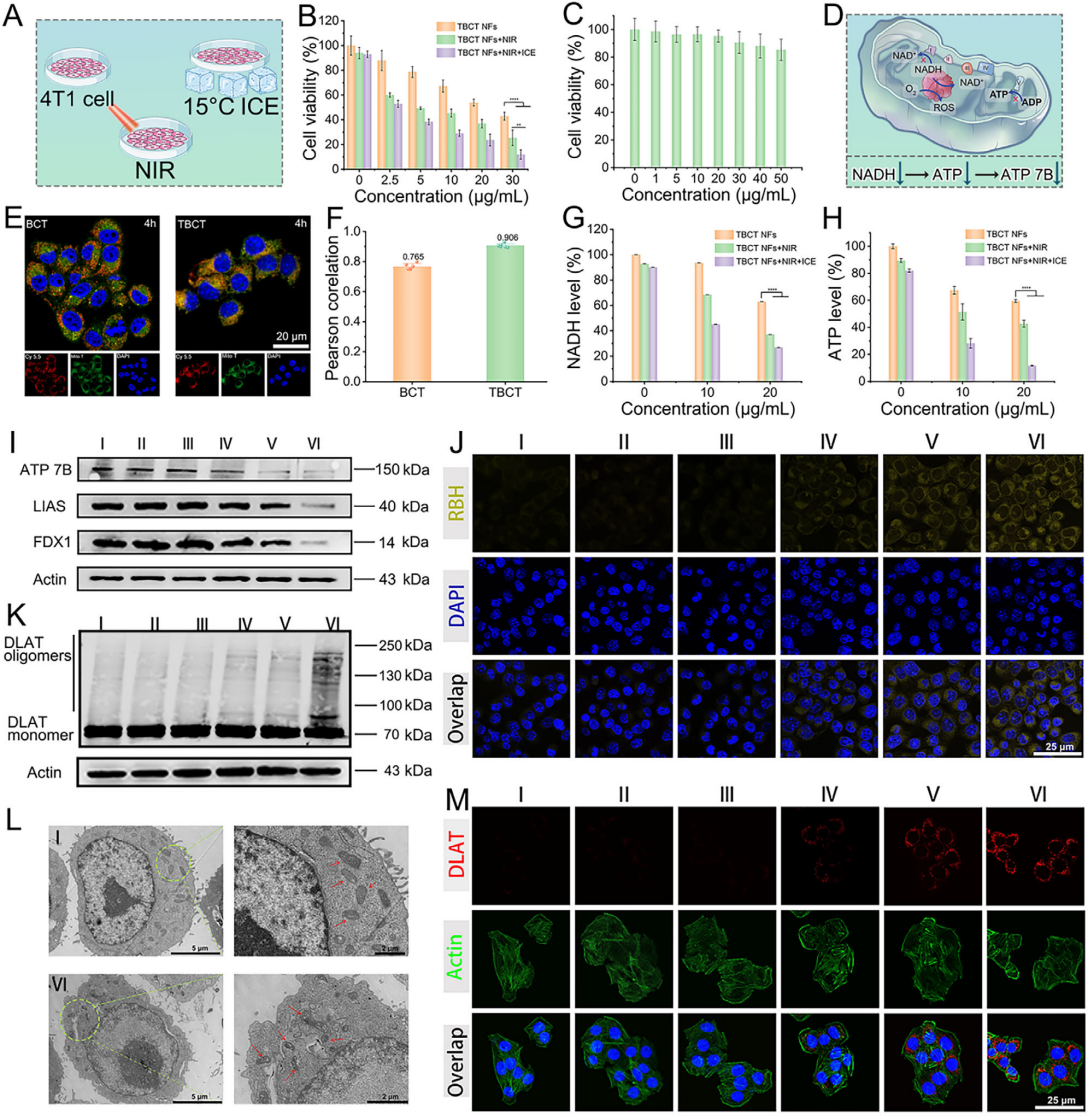
In vitro evaluation of cuproptosis properties. (A) Schematic of the thermal cycling procedure mediated by successive 808 nm laser heating and ice‐bath cooling. (B) Cytotoxicity of 4T1 cells was evaluated following treatment with TBCT NFs at various concentrations and under different conditions. (C) Cell viability of L929 cells after incubation with TBCT NFs for 24 h. (D) Schematic diagram of the mechanism for ATP7B suppression. (E) CLSM images of the mitochondrial localization of Cy 5.5‐labeled BCT NFs and TBCT NFs. (F) Statistical analysis of mitochondrial co‐localization (Pearson's correlation coefficient, PCC). (G) Measurement of intracellular NADH levels across different treatment groups. (H) Cellular ATP level in 4T1 cells with different treatments. (I) Western blotting analysis on the expression levels of cuproptosis‐related proteins (LIAS, FDX1, and ATP7B) in 4T1 cells treated with (I) Control, (II) NIR, (III) NIR+ICE, (IV) TBCT, (V) TBCT+NIR, (VI) TBCT+NIR+ICE. (J) CLSM of 4T1 cells stained with RBH. (K) Western blotting analysis on the expression levels of DLAT. (L) Bio‐TEM examination of mitochondria in 4T1 cells under control and TBCT+NIR+ICE conditions. (M) CLSM of 4T1 cells stained with DLAT. Data are shown as mean ± SD (n = 6), *p*‐values were calculated using one‐way ANOVA with Tukey's post‐hoc test, *p < 0.05, **p < 0.01, ***p < 0.001, and ****p < 0.0001.

We have proved in the previous section that our TBCT NFs can efficiently deplete NADH by oxidating it into NAD^+^. Such ability, when occurring within mitochondria, would significantly inhibit ATP biosynthesis (Figure 4D). We now examined the ability of TBCT NFs to cause ATP outage and the sequential ATP7B dysfunction. The TBCT NFs were functionalized with TPP, a delocalized lipophilic cation widely recognized for conferring mitochondrial targeting. To validate their mitochondrial localization, we performed confocal laser scanning microscopy (CLSM) using Cy5.5‐labeled TBCT NFs. Their colocalization with mitochondria, pre‐stained with MitoTracker Green, was assessed over multiple time points. These results showed that Cy5.5‐labeled TBCT NFs exhibited prominent mitochondrial colocalization by 4 h post‐incubation (Figure S10). To further confirm the TPP‐driven targeting specificity, a control experiment was conducted using non‐mitochondria‐targeted NFs (BCT NFs). The CLSM images revealed superior mitochondrial accumulation for TBCT NFs compared to the BCT NFs (Figure 4E). This colocalization was quantitatively analyzed by calculating the Pearson's correlation coefficient (PCC). The PCC value for TBCT NFs (0.906 ± 0.01) was significantly higher than that for BCT NFs (0.765 ± 0.02) (Figure 4F), quantitatively confirming the enhanced mitochondrial targeting efficiency conferred by TPP modification.

We next measured intracellular NADH levels after 12 h of thermal cycling. Compared with the control group, NADH was depleted in a concentration‐dependent manner (Figure 4G), indicating substantial NADH consumption driven by the thermoelectric effect. Consistently, ATP levels in TBCT NFs‐treated cells decreased markedly compared with the control group, with a reduction of approximately 60% at 20 µg mL^−1^ (Figure 4H). To further verify the effect of TBCT NFs on ATP after thermal cycling, we performed an adenosine‐based ATP rescue experiment (Figure S11a). In the TBCT NFs+NIR+ICE group, intracellular ATP levels decreased by approximately 60%, supplementation with 300 µM adenosine restored ATP levels to 66.6% of the control, indicating that adenosine treatment effectively replenished intracellular ATP. These results demonstrate that the thermoelectric effect of TBCT NFs effectively consumes ETC substrates, thereby significantly suppressing ATP production.

More importantly, we assessed the functional state of ATP7B in such ATP‐scarcity situation. ATP7B expression was markedly downregulated in the TBCT NFs+NIR+ICE(VI) group (Figure 4I and Figure S24a). This downregulation likely impairs copper efflux, disrupts copper homeostasis, and exacerbates cuproptosis. To determine whether ATP7B inhibition affects intracellular copper levels, we employed the Rhodamine B hydrazide (RBH) fluorescent probe to monitor changes in copper ion content (Figure 4J). As depicted in the figure, the fluorescence intensity in the TBCT NFs group (IV) was significantly higher than that in the control groups (I, II, and III). Moreover, the TBCT NFs+NIR+ICE group (VI) exhibited stronger fluorescence compared to both the TBCT NFs group (IV) and the TBCT NFs+NIR group (V), indicating that enhanced thermal gradient via ICE cooling effectively suppresses copper efflux, leading to increased intracellular copper accumulation. To further quantify intracellular copper content and compare the effects across different treatment groups, we performed ICP‐OES. These results (Figure S12) confirmed that the copper level in the TBCT NFs group (IV) was significantly higher than that in the control groups (I, II, and III). Consistent with the fluorescence data, the TBCT NFs+NIR+ICE group (VI) showed the highest copper content, significantly exceeding that in both the TBCT NFs group (IV) and the TBCT NFs+NIR group (V). These quantitative measurements robustly support the conclusion that the combined treatment strategy potently enhances intracellular copper retention.

To further establish the causal relationship between ATP depletion and copper retention, we monitored the temporal sequence of these events at 0, 0.5, 1, 2, 4, and 8 h after irradiation. The results revealed a clear cascade: ATP depletion occurred as early as 0.5 h (Figure S11b), followed by impaired copper efflux at 1–2 h (Figure S13a), ultimately leading to intracellular copper accumulation. This causal relationship was subsequently validated through adenosine‐based ATP rescue experiments, adenosine supplementation restored ATP levels and reduced copper retention. Moreover, the P‐type ATPase inhibitor Na_3_VO_4_ abolished this rescue effect, confirming that ATP acts specifically through ATP7B (Figure S13b). Collectively, these findings unequivocally establish the ATP depletion‐driven blockade of the ATP7B‐copper efflux axis as a causally validated unique mechanistic cascade.

To verify cuproptosis induction, cells were treated with the Cu‐specific chelator tetrathiomolybdate (TTM). The dose‐dependent rescue of cell viability by TTM (0–20 µM, 12 h) confirmed intracellular copper‐dependent cell death (Figure S14). Copper ions entering cells bind to lipoylated mitochondrial proteins such as DLAT, inducing their toxic oligomerization and subsequent cell death, highlighting DLAT critical role in cuproptosis. Consistent with this mechanism, Figure 4K demonstrates prominent DLAT oligomerization in the VI group (Figure S24b). Furthermore, immunofluorescence staining confirmed significant differences in DLAT oligomerization across treatment conditions (Figure 4M). Cellular morphological changes in control and VI groups were examined by transmission electron microscopy (Figure 4L). Mitochondria in the VI‐treated group exhibited severe damage, characterized by condensation and distinct cristae loss or reduction, indicative of cuproptosis. These results demonstrate that TBCT NFs impair ATP7B activity via the thermoelectric effect, thereby inhibiting copper efflux and potentiating cuproptosis.

### 2.5 In Vitro ICD and Apoptosis of TBCT NFs

Cuproptosis has been reported to promote the release of damage‐associated molecular patterns (DAMPs) from tumor cells, a key process in triggering immunogenic cell death (ICD) [5, 6, 7]. Representative DAMPs include adenosine triphosphate (ATP), high‐mobility group box 1 (HMGB1), and calreticulin (CRT). Their extracellular release activates dendritic cells (DCs), initiates innate immune responses, and ultimately contributes to ICD (Figure 5A). To investigate whether TBCT NFs enhance cuproptosis through the thermoelectric effect to induce DAMP release after 12 h of treatment, we measured extracellular ATP and HMGB1 levels. As shown in Figure 5B,C, the group treated with TBCT NFs+NIR+ICE (VI) for 12 h exhibited significantly elevated ATP and HMGB1 release compared with the control, indicating a high potential for ICD induction. Furthermore, immunofluorescence staining revealed pronounced CRT exposure on the cell surface in group VI after 12 h, confirming the effective translocation of this signal (Figure 5D). To establish the causal link between cuproptosis and DAMP release, we included a cuproptosis‐inhibited control group (VII). TTM treatment markedly suppressed the release of HMGB1 and the exposure of CRT, confirming that DAMP exposure is specifically dependent on cuproptosis rather than nonspecific cell damage. Collectively, these data demonstrate that TBCT NFs effectively induce ICD through cuproptosis‐driven DAMP release, providing a mechanistic basis for subsequent immune activation.

**FIGURE 5:**
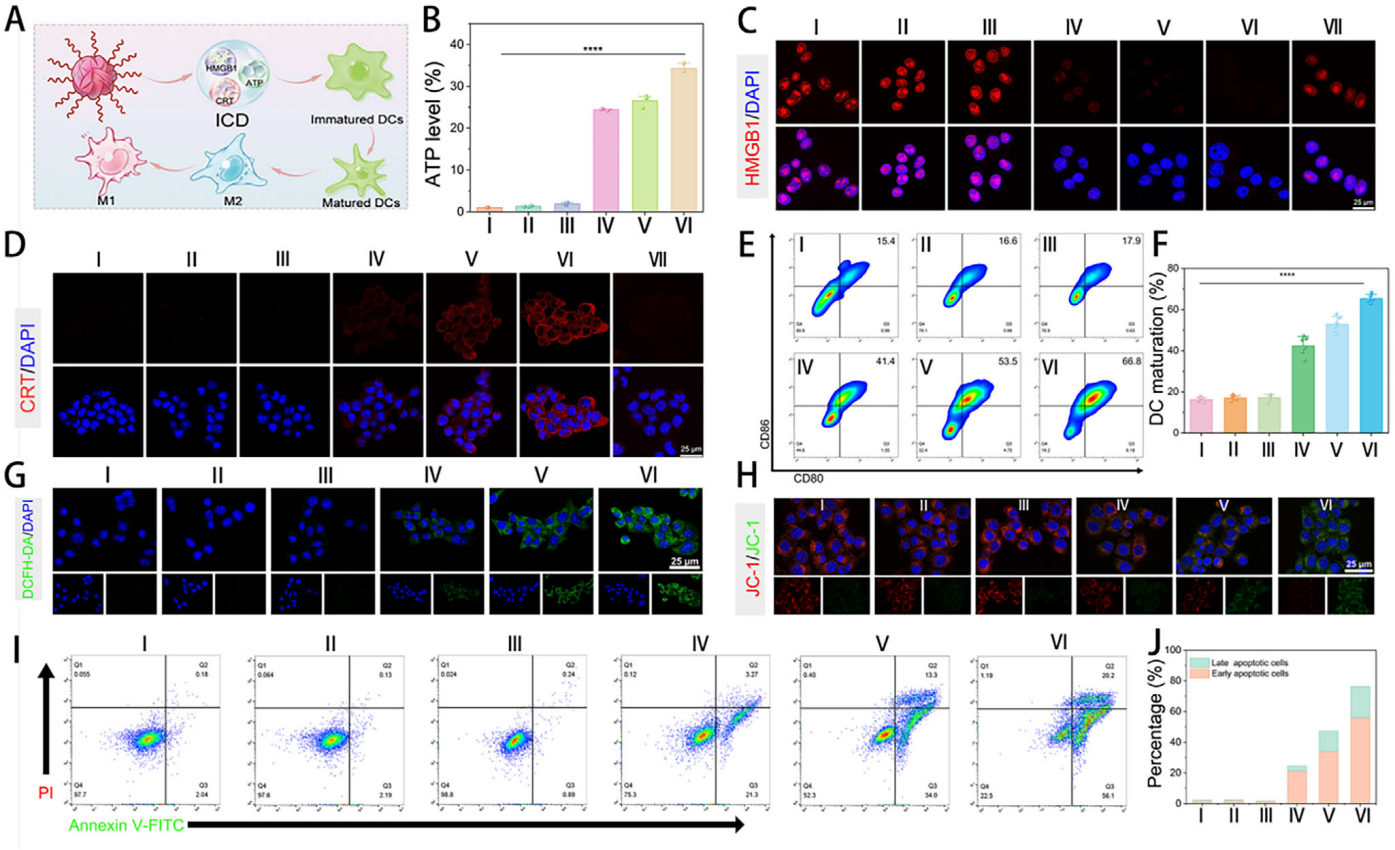
In vitro evaluation of immunotherapy properties and apoptosis. (A) Schematic of TBCT‐activated immunotherapy. (B) Extracellular ATP levels in 4T1 cells subjected to various treatments. Immunofluorescence analysis of (C) HMGB1 and (D) CRT under different treatment conditions. (I) Control, (II) NIR, (III) NIR+ICE, (IV) TBCT, (V) TBCT+NIR, (VI) TBCT+NIR+ICE, (VII) TBCT+NIR+ICE+TTM. (E) Flow cytometry was used to detect mature DCs (CD11c+, CD80+, and CD86+). (F) Quantitative analysis of the maturity of DCs tested by flow cytometry. Data are shown as mean ± SD (n = 6), *p*‐values were calculated using one‐way ANOVA with Tukey's post‐hoc test, *p < 0.05, **p < 0.01, ***p < 0.001, and ****p < 0.0001. (G) ROS level after various treatments. (H) JC‐1 staining for assessing mitochondrial membrane potential. (I) Flow cytometric analysis of apoptosis induction and (J) Quantitative assessment of apoptotic rates in 4T1 cells subjected to the indicated treatments.

To validate the antigen‐presenting capability of TBCT NFs at the cellular level, we employed DC2.4 dendritic cells as an in vitro model. 4T1 tumor cells were first treated with TBCT NFs under different conditions for 12 h, and subsequently cocultured with DC2.4 cells for another 12 h. The maturation status of DC2.4 cells was then evaluated by flow cytometry based on specific surface markers (Figure 5E,F). Compared with the group treated with TBCT NFs + NIR (V), DC maturation was further enhanced in the group receiving TBCT NFs + NIR + ICE (VI).

Besides triggering ICD, cuproptosis typically induces oxidative stress in tumor cells. Potent oxidative stress further triggers apoptotic cell death, thereby contributing to significant antitumor efficacy. To assess intracellular ROS generation by TBCT NFs, we measured ROS production in 4T1 cells using 2',7'‐dichlorodihydrofluorescein diacetate (DCFH‐DA) (Figure 5G). The TBCT NFs+NIR+ICE (VI) group exhibited significantly stronger green fluorescence compared to controls, suggesting enhanced ROS generation associated with the thermoelectric effect and Fenton‐like reactions. The •O_2_^−^ levels in treated 4T1 cells were also evaluated using dihydroethidium (DHE) as a fluorescent probe (Figure S15). The mitochondrial membrane potential (Δ*Ψ*_m_), a key indicator of early apoptosis, was monitored using the JC‐1. This assessment was motivated by the central role of mitochondria in the apoptotic process. Control groups (I, II, III) exhibited intense red fluorescence, indicating high Δ*Ψ*_m_. Notably, the TBCT NFs (IV) group showed reduced green fluorescence, while both the TBCT NFs+NIR (V) and VI groups displayed intense green fluorescence (Figure 5H). This shift suggests that laser irradiation combined with cold‐to‐hot transformation potentiates TBCT NFs‐induced mitochondrial membrane damage. Furthermore, anti‐tumor efficacy was assessed using Calcein AM and propidium iodide (PI) double‐staining (Figure S16). Collectively, these results indicate that TBCT NFs, under photothermal therapy and thermoelectric effects, effectively induce apoptosis.

Apoptosis induction by TBCT NFs was assessed using annexin V‐FITC and PI double staining (Figure 5I,J). The VI group exhibited a significantly higher apoptotic rate (76.3%) compared to the control (2.22%), NIR (2.32%), NIR+ICE (1.92%), TBCT NFs (24.57%), and TBCT NFs+NIR (47.3%) groups. These results demonstrate that the TBCT NFs+NIR+ICE (VI) combination robustly induced apoptosis. Bcl‐2 (anti‐apoptotic) and Bax (pro‐apoptotic) are key regulators of apoptosis; their expression ratio critically influences apoptotic commitment and progression. Western blot analysis (Figure S17 and S24c) revealed significantly decreased Bcl‐2 expression and increased Bax expression in the IV, V, and VI groups compared to controls (I, II, III). These findings demonstrate that TBCT NFs can also induce apoptosis for additional anti‐tumor efficacy, and this apoptotic effect can be potentiated by the thermoelectric and photothermal effects.

### 2.6 In Vivo Therapeutic Performance of TBCT NFs

The in vivo biodistribution of TBCT NFs was evaluated in 4T1 tumor‐bearing mice using small‐animal in vivo imaging (Figure 6A). Cy5.5‐labeled TBCT NFs were intravenously administered via tail vein injection. Fluorescence intensity was monitored at 0, 1, 3, 6, 12, 24, and 48 h post‐injection. Tumor fluorescence intensity progressively increased over time, peaking at 12 h, followed by a gradual decrease. Ex vivo imaging of major organs (heart, liver, spleen, lung, kidney) and tumors at 48 h revealed significantly higher fluorescence intensity in tumor tissue compared to all other organs. This demonstrates preferential tumor accumulation and prolonged retention of TBCT NFs. To assess the distribution of TBCT NFs in the organism, we injected TBCT NFs intravenously and analyzed the Cu and Te content in main organs and tumors. The concentrations of both elements in the tumor increased over time, peaking at 12 h post‐injection at 14.3 ± 0.8% ID g^−1^ for Cu and 8.1 ± 0.2% ID g^−1^ for Te (Figure S18).

**FIGURE 6:**
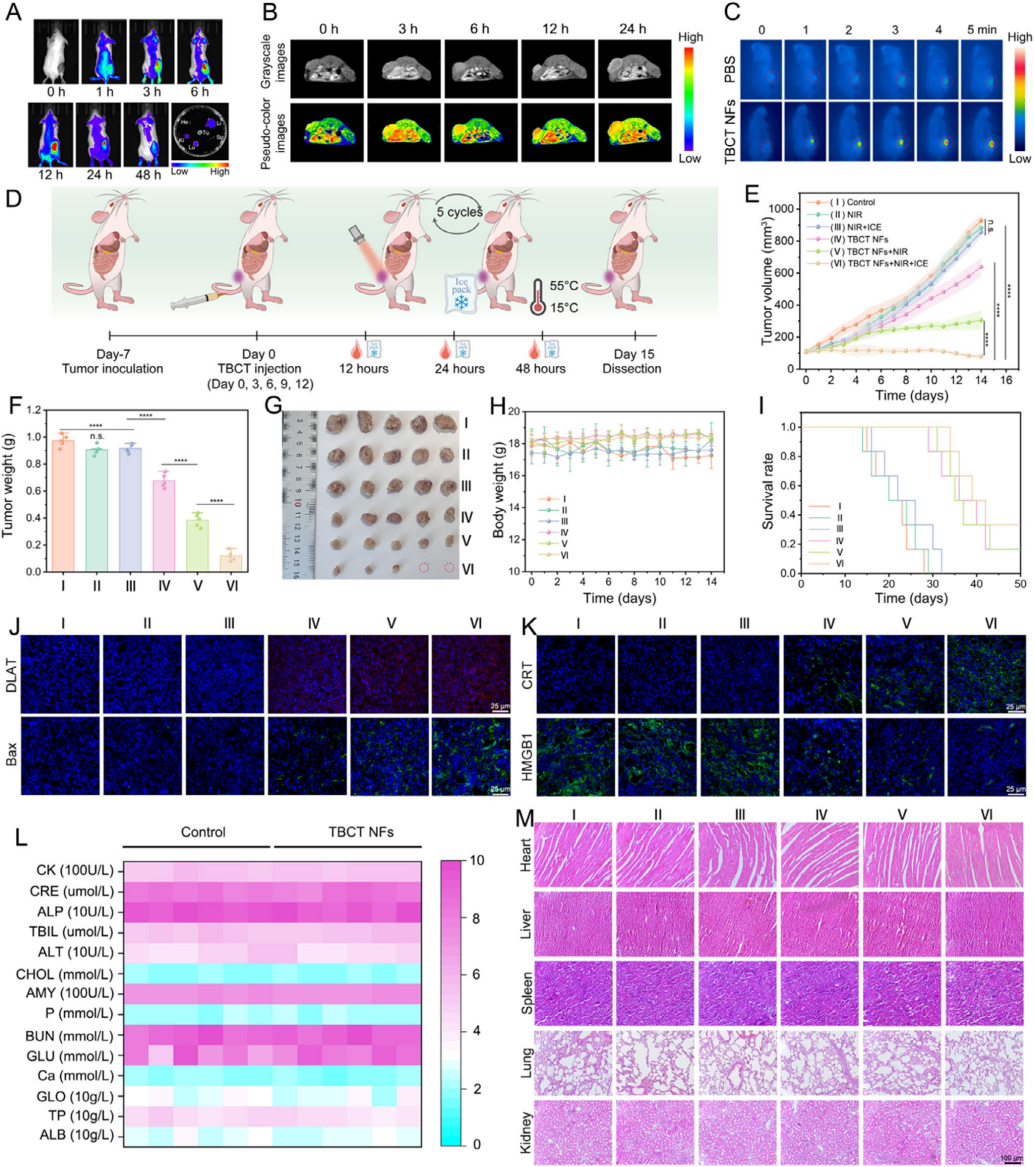
In vivo therapeutic performance of TBCT NFs. (A) Fluorescence imaging of TBCT NF distribution in 4T1 tumor‐bearing mice, and distribution in tumor and major organs ex vivo at 24 h. (B) Temporal evolution of *T*_1_‐weighted MRI signal in 4T1 tumors following the intravenous injection of TBCT NFs. (C) Thermal imaging of 4T1 tumor models post‐treatment via tail vein under 808 nm laser irradiation. (D) Schematic illustration of animal therapy by TBCT NFs. (E) Tumor volume, (F) tumor weights, and (G) photographs of excised tumors after treatment. (H) Body weight variations and (I) Kaplan–Meier analysis of survival rates in 4T1 tumor‐bearing mice following various treatments. (J) Immunofluorescent analysis of DLAT and Bax expression in tumor tissues post‐treatment. (K) Expression of HMGB1 and CRT in tumor regions by immunofluorescent. (L) Assessment of hematological and biochemical parameters in mice following a 14‐day intravenous administration of PBS or TBCT NFs. (M) H&E staining of normal tissues obtained from different treatment groups. Data are shown as mean ± SD (n = 5), *p*‐values were calculated using one‐way ANOVA with Tukey's post‐hoc test, *p < 0.05, **p < 0.01, ***p < 0.001, and ****p < 0.0001.

Magnetic resonance imaging (MRI), a safe, non‐invasive, and high‐resolution technique, is widely employed in medicine. Previous studies indicate the potential of copper‐based nanoparticles as MRI contrast agents, suggesting TBCT NFs possess similar capability. Building on these findings, in vitro assessment revealed that the *T*_1_ signal intensity of TBCT NFs increased linearly with concentration (Figure S19), exhibiting a longitudinal relaxivity (r_1_) of 3.992 mM^−1^s^−1^. Subsequently, the time‐dependent in vivo MRI contrast enhancement of TBCT NFs was evaluated in tumor‐bearing mice following intravenous administration at 0, 3, 6, 12, and 24 h. *T*_1_‐weighted MR signal intensity in the tumor region increased progressively over time, reaching maximum enhancement at 12 h post‐injection (Figure 6B). This pronounced and sustained tumor signal enhancement demonstrates effective accumulation of TBCT NFs within the tumor tissue, primarily attributed to the enhanced permeability and retention (EPR) effect. Furthermore, intratumoral temperature changes were monitored using thermal imaging at various time points post‐injection under 808 nm irradiation (1.0 W cm^−2^) (Figure 6C). Tumor temperature increased rapidly upon irradiation. Notably, the TBCT NFs+NIR group reached a significantly higher temperature of 56.5°C within 5 min compared to the PBS+NIR control group (38.4°C). This substantial photothermal effect demonstrates the excellent in vivo photothermal conversion capability of TBCT NFs.

To assess the in vivo antitumor efficacy of TBCT NFs, a xenograft model was established through subcutaneous implantation of 4T1 cells (4 × 10^6^) into the right flank of female BALB/c mice. Thirty tumor‐bearing mice were randomly divided into six groups (n = 5 per group) and treated as follows (Figure 6D): (I) PBS control (no treatment); (II) NIR laser irradiation only (808 nm irradiation, 1.0 W cm^−2^); (III) Cycled thermal intervention: ice‐pack cooling applied during 808 nm irradiation (1.0 W cm^−2^) at 12, 24, and 48 h (5 cycles per session); (IV) TBCT NFs only (15 mg kg^−1^); (V) TBCT NFs (15 mg kg^−1^) + NIR laser (808 nm irradiation, 1.0 W cm^−2^); (VI) TBCT NFs (15 mg kg^−1^) + cycled thermal intervention (808 nm irradiation + ice‐pack cooling at 12, 24, and 48 h; 5 cycles per session). For groups (IV)–(VI), mice received tail vein injections of each treatment (15 mg kg^−1^) every 3 days starting from Day 0. For thermal cycling (Groups III and VI), ice packs were applied immediately after laser exposure to rapidly reduce tissue temperature to 15°C, maximizing thermal contrast to enhance therapeutic efficacy. After 14 days of treatment, Group VI (TBCT NFs + NIR + ICE) exhibited a significantly reduced tumor volume of 79 ± 19 mm^3^, compared with 928 ± 34 mm^3^ in the control group (Figure 6E), indicating potent antitumor efficacy. Tumor mass analysis further revealed a marked decrease in tumor weight in Group VI (0.12 ± 0.03 g) relative to the control group (0.97 ± 0.05 g) (Figure 6F). Consistent with these findings, macroscopic photographs of the excised tumors visually confirmed substantially enhanced therapeutic outcomes under cold‐to‐hot transformation conditions (Figure 6G). Furthermore, body weight monitoring over the 14‐day assessment period showed no significant fluctuations across all groups (Figure 6H), indicating minimal systemic toxicity of TBCT NFs. Crucially, the TBCT NFs+NIR+ICE (Group VI) combinatorial therapy group exhibited the longest survival duration without observable tumor recurrence, confirming markedly improved survival rates in tumor‐bearing mice (Figure 6I).

In summary, the therapeutic efficacy of TBCT NFs was significantly enhanced following thermal cycling treatment. To elucidate the mechanism underlying the synergistic therapy, we performed immunofluorescence staining on tumor sections to evaluate changes in relevant proteins across different treatment groups (Figure 6J). The occurrence of cuproptosis was initially assessed based on DLAT protein expression. Compared to the control group (Groups I, II, and III), DLAT levels progressively increased in the TBCT NFs (IV), TBCT NFs +NIR (V), and TBCT NFs +NIR +ICE (VI) groups, indicating a gradual enhancement of cuproptosis. Bax, a pro‐apoptotic protein, was analyzed to evaluate apoptosis induction. IF results revealed markedly elevated Bax levels in the TBCT NFs +NIR +ICE group (VI), signifying apoptosis activation.

HMGB1 and CRT serve as hallmark proteins of ICD (Figure 6K). Assessment of HMGB1 fluorescence intensity demonstrated a progressive decrease across the TBCT NFs (IV), TBCT NFs +NIR (V), and TBCT NFs +NIR +ICE (VI) groups. This pattern suggests gradual HMGB1 release into the extracellular space, potentially enhancing immune cell recruitment. Conversely, CRT fluorescence intensity progressively increased, indicative of CRT translocation to the cell membrane, thereby augmenting immunogenicity. Collectively, the alterations in HMGB1 and CRT levels provide preliminary evidence for ICD induction.

To evaluate the biocompatibility of TBCT NFs, healthy BALB/c mice received tail vein injections, followed by complete blood count (CBC) and blood biochemical analyses. No significant differences were observed in any hematological or biochemical biomarkers between the treated and control groups (Figure 6L). Hemolysis assays were performed to further evaluate the biocompatibility of TBCT NFs, which exhibited minimal hemolysis across different concentration gradients (Figure S20). Furthermore, hematoxylin and eosin (H&E) staining revealed no apparent inflammation or injury in the major organs of mice from any group 15 days post‐treatment (Figure 6M). These findings collectively demonstrate the favorable biosafety profile of TBCT NFs.

### 2.7 In Vivo Immunotherapy Effects of TBCT NFs

To evaluate the immunotherapeutic efficacy of TBCT NFs, we adopted the same in vivo treatment regimen as described above and subsequently analyzed the expression profiles of distinct immune cell populations by flow cytometry. Following TBCT NFs treatment, the maturation ratio of DCs in lymph nodes significantly increased (Figure 7A,B). Notably, a significant difference was observed between the TBCT NFs +NIR+ICE group and the TBCT NFs +NIR group, suggesting enhanced antigen‐presenting capacity and immune system activation after thermal cycling. Compared to the control group, TBCT NFs treatment significantly reduced the proportion of myeloid‐derived suppressor cells (MDSCs) (Figure 7C,D), indicating an alleviated immunosuppressive tumor microenvironment and improved immunotherapeutic efficacy. Furthermore, we quantified tumor‐infiltrating CD3^+^ T cells and CD8^+^ T cells. The control group exhibited 28.2% CD3^+^ T cells (Figure 7E,F), while the TBCT NFs group and TBCT NFs +NIR+ICE group showed 34.8% and 48.8%, respectively. This progressive increase demonstrates that thermal cycling significantly enhances the functionality of TBCT NFs, promoting T cell infiltration. Concurrently, the number of CD8^+^ T cells in treated groups increased significantly (Figure 7G,H), with the TBCT NFs +NIR +ICE group showing maximal enhancement. This indicates that TBCT NFs effectively stimulated tumor‐specific immune responses and promoted T‐cell differentiation.

**FIGURE 7:**
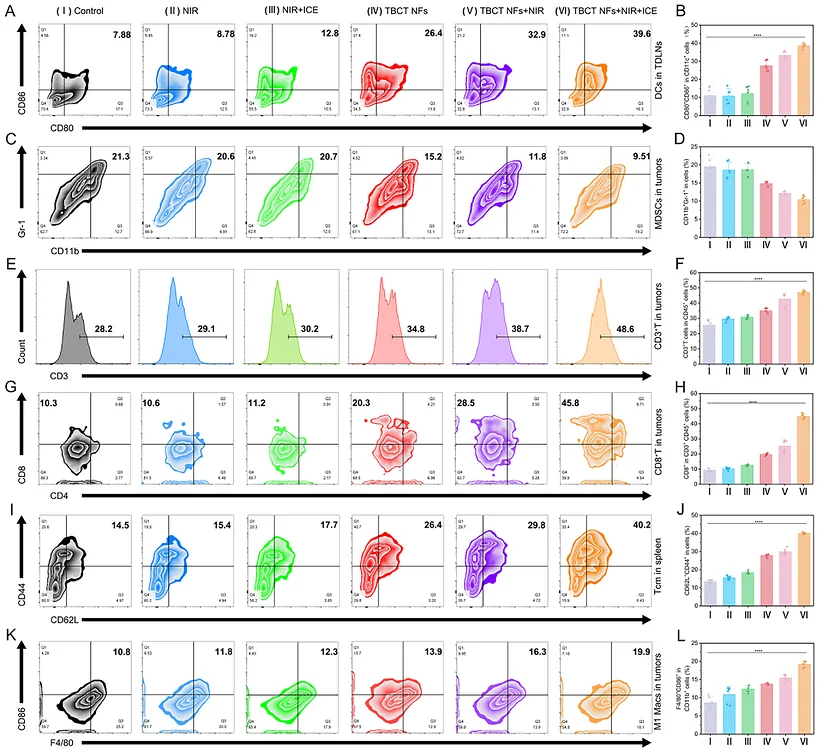
In vivo immune assessment of TBCT NFs. (A) DC maturation rate determined by flow cytometry. (B) Statistical analysis of maturation rates of DCs. (C) MDSCs determined by flow cytometry. (D) Statistical analysis of MDSCs. (E) CD3^+^ T cells in the tumor. (F) Statistical analysis of CD3^+^ T cells in the tumor. (G) CD8^+^ CD4^+^ T cells in the tumor. (H) Statistical analysis of CD8^+^ T cells in the tumor. (I) Flow cytometric analysis of central memory T cells (T_cm_) following various treatments. (J) Statistical analysis of T_cm_ cells. (K) M1 TAMs in tumors determined by flow cytometry. (L) Statistical analysis of maturation rates of M1 macrophages. Data are shown as mean ± SD, n = 6, ns. indicated no significance, *p*‐values were calculated using one‐way ANOVA with Tukey's post‐hoc test; *p < 0.05, **p < 0.01, ***p < 0.001, and ****p < 0.0001.

We further assessed T‐cell differentiation in the spleen. Post‐treatment, both CD4^+^ and CD8^+^ T‐cell populations markedly expanded (Figure S21). Notably, the proportion of central memory T cells (T_cm_) within these subsets significantly increased. To directly compare T_cm_ cell quantities, we show total splenic T_cm_ counts (Figure 7I,J). TBCT NFs treatment substantially elevated T_cm_ numbers, with the TBCT NFs +NIR+ICE group exhibiting the most pronounced effect. These data demonstrate that the thermoelectric effect potently enhances T_cm_ differentiation in the spleen, suggesting sustained immunologic memory and microenvironment remodeling.

As innate immune effectors, macrophages play pivotal roles in pathogen response, inflammation modulation, and tissue repair. Whether these cells polarize into pro‐inflammatory (M1) or anti‐inflammatory (M2) phenotypes depends on the microenvironment, and M2 polarization promotes tumor progression. Flow cytometric analysis revealed that treatment groups exhibited significantly higher M1 macrophage proportions versus controls (Figure 7K,L), while M2 proportions progressively decreased (Figure S22). This indicates TBCT NFs effectively reprogram macrophages from protumor M2 toward antitumor M1 phenotypes. These results demonstrate that TBCT NFs increase antitumor immune cells while reducing immunosuppressive populations. The enhanced immunotherapeutic efficacy under thermoelectric performance validates the novel application of thermoelectric materials in tumor immunotherapy.

### 2.8 RNA‐Sequencing Analysis of Anti‐Cancer Mechanism of TBCT NFs

Following treatment of 4T1 cells with PBS or with TBCT NFs +NIR+ICE for 12 h, RNA sequencing was performed to elucidate the transcriptomic mechanisms underlying the synergistic therapy of cuproptosis and immunogenic cell death (ICD). Comparative analysis between the TBCT NFs +NIR+ICE group and control group identified 26,816 differentially expressed genes (DEGs), including 1250 significantly upregulated and 4,678 significantly downregulated genes (Figure 8A). KEGG enrichment analysis revealed that TBCT NFs +NIR+ICE treatment modulated key immune‐related pathways: IL‐17 signaling, Toll‐like receptor signaling, Th17 cell differentiation, and chemokine signaling (Figure 8B). These pathways critically regulate immune responses, indicating strong relevance to immunotherapy mechanisms. Functional categorization of DEG‐associated pathways and corresponding enriched gene counts are presented in Figure 8C. Gene Ontology (GO) analysis further delineated biological processes, cellular components, and molecular functions affected by DEGs, with bar heights indicating upregulated/downregulated gene numbers per term (Figure 8D).

**FIGURE 8:**
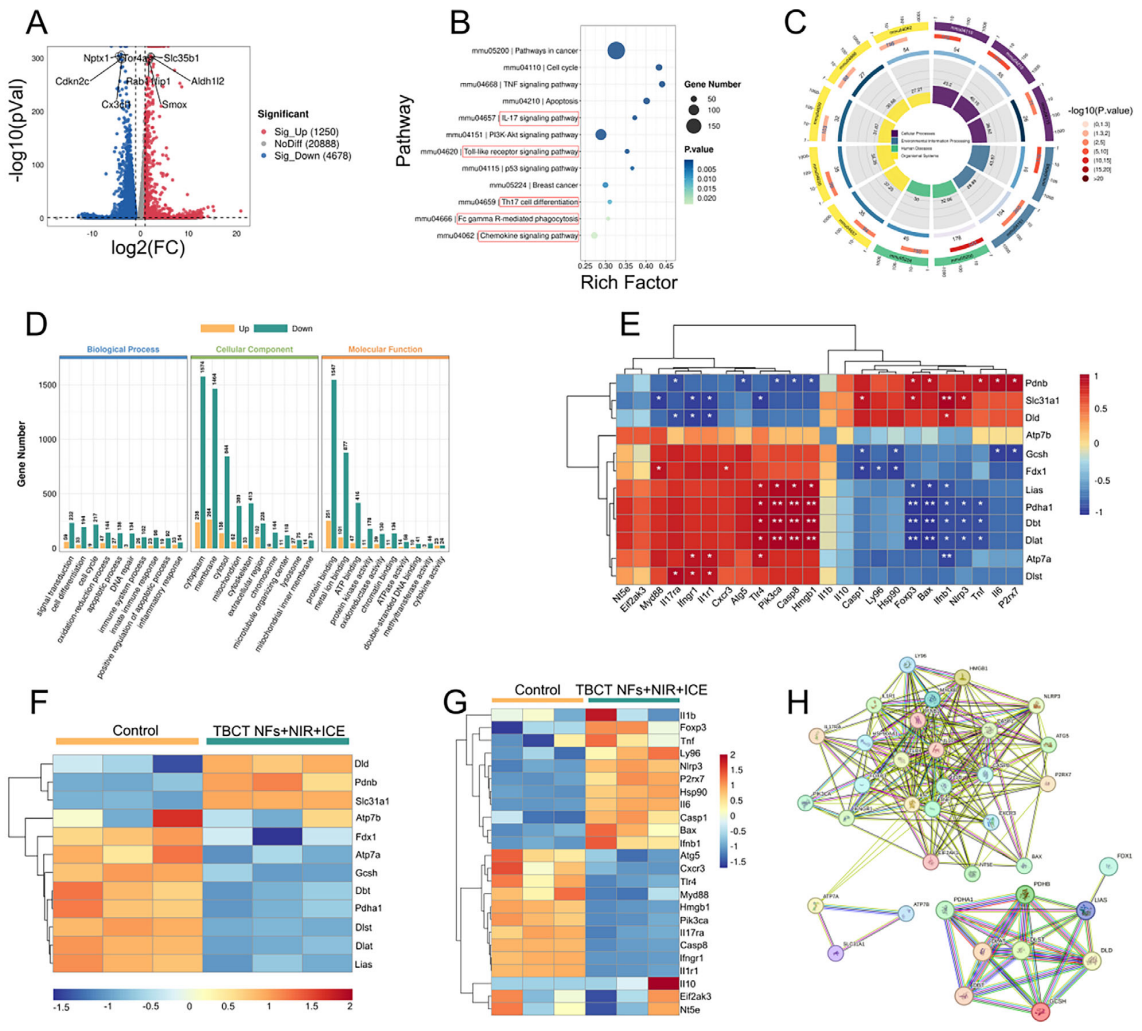
Transcriptional analysis of anti‐tumor mechanism induced by TBCT NFs+NIR+ICE. (A) Volcano plot of the differentially expressed genes (DEGs) between the control and TBCT NFs+NIR+ICE treatment group. (B) KEGG pathway enrichment analysis. (C) Circos plots of KEGG analysis after different treatments. (D) GO enrichment analysis revealed the most significantly enriched biological processes for upregulated and downregulated genes. (E) Cluster heatmap of the DEGs associated with cuproptosis and ICD between TBCT NFs+NIR+ICE and control groups. (F) Cluster heatmap of the DEGs associated with cuproptosis between TBCT NFs+NIR+ICE and control groups. (G) Cluster heatmap of the DEGs associated with ICD between TBCT NFs+NIR+ICE and control groups. (H) Functional association networks of variably expressed cuproptosis and ICD proteins.

To evaluate crosstalk between cuproptosis and immunogenic cell death (ICD), we generated expression heatmaps of cuproptosis‐related genes (Figure 8F) and ICD‐related genes (Figure 8G). To more intuitively present the normalized expression (FPKM) of key cuproptosis‐related genes, we additionally provided bar plots with statistical analysis (Figure S23). Spearman correlation analysis demonstrated significant associations between these gene sets (Figure 8E). Protein‐protein interaction (PPI) networks constructed via STRING database confirmed functional linkages between cuproptosis and ICD pathways (Figure 8H).

## 3 Conclusions

In this study, we fabricated thermoelectric TBCT NFs to disrupt the ATP energy supply for the copper transporter ATP7B, thereby enhancing cuproptosis and achieving synergistic immunotherapy for anti‐tumor purposes. Our design centers on disrupting the mitochondrial electron transport chain by leveraging the TBCT NFs excellent thermoelectric effect. To this end, the TBCT NFs was fabricated with the following considerations: (1) A Bi^3+^ doping strategy was adopted to significantly improve the Seebeck coefficient of the Cu_2−x_Te base material. (2) A unique NFs morphology was engineered to provide a larger surface area for efficient temperature gradient generation under laser irradiation. (3) The TBCT NFs was decorated with a mitochondria‐targeting TPP moiety to concentrate its function within mitochondria. We demonstrated that the resulting TBCT NFs effectively impeded ATP biosynthesis, causing ATP7B dysfunction and resulting in significant intracellular Cu^+^ accumulation.

Compared with reported nanoplatforms, our TBCT NFs exhibit an ultralow thermal conductivity of 0.46 W m^−1^ K^−1^, lower than that of typical bio‐thermoelectric materials such as Cu_2_SnS_3_ [44]. Although the Seebeck coefficient (21 µV K^−1^) and figure of merit ZT (0.041) are moderate relative to top‐tier bulk thermoelectric (Cu_2_Se) [45], the integration of photothermal conversion and thermoelectric catalysis within a single mitochondria‐targeted nanoparticle generates a locally amplified energy crisis, resulting in impressive ATP depletion of 88 ± 5% compared with the control group. This far exceeds that achieved by single‐mode therapies or previous cuproptosis systems [31, 32, 33]. Correspondingly, intracellular copper increases 2.1‐fold, DLAT oligomerization increases 4.0‐fold over the untreated control, and the CRT fluorescence intensity of the TBCT NFs+NIR+ICE group is ten‐fold higher than that of the blank group. All these values surpass those reported for existing systems [46, 47, 48]. These comparisons demonstrate the excellent efficiency of our integrated platform in inhibiting ATP synthesis.

Another contribution of our work is the identification of ATP7B dysfunction as a key mediator linking ATP depletion to potent cuproptosis. Mechanistically, we showed that thermoelectric catalysis‐induced ATP depletion blocked the ATP7B‐copper efflux axis, thereby driving intracellular copper retention and subsequent cuproptosis. More importantly, we showed that this enhanced cuproptosis promoted prominent DAMP release, which subsequently triggered ICD to activate anti‐tumor immunity. We further verified the functional crosstalk between cuproptosis and immunotherapy via RNA sequencing, which revealed significant correlations between cuproptosis‐related and ICD‐related genes following treatment with thermoelectric TBCT NFs. Collectively, our study exemplifies the use of advanced thermoelectric nanomedicine to achieve a cuproptosis‐immunotherapy cascade anti‐tumor effect by perturbing cancer cell energy metabolism.

## 4 Experimental Section

### 4.1 Synthesis of PAA‐Cu/Bi Nanospheres (NSs) and TPP‐Bi‐Cu_7_Te_4_ Nanoflowers (TBCT NFs)

Aqueous ammonia (2 M, 150 µL) and polyacrylic acid (PAA, 0.2 g mL^−1^, 100 µL) were dissolved in deionized water (10 mL) under magnetic stirring (30 min, RT). Isopropanol (60 mL) was added dropwise, inducing a phase transition from a clear solution to milky white dispersion, yielding well‐dispersed PAA NSs. Subsequently, Cu(NO_3_)_2_·3H_2_O (30 mg in 1 mL H_2_O) and Bi(NO_3_)_3_ (30 mg in 5 mL ethylene glycol) were sequentially introduced. After 12 h stirring (RT), the solution color shifted to pale blue, affording PAA‐Cu/Bi NSs.

PAA‐Cu/Bi NSs (20 mg) and K_2_TeO_3_ (20 mg) were dispersed in a H_2_O/isopropanol mixed solvent (1:1 v/v, 10 mL). After 1 h stirring (RT), the reaction proceeded at 200°C for 12 h, yielding Bi‐Cu_7_Te_4_ NFs.

### 4.2 Synthesis of TPP‐Bi‐Cu_7_Te_4_ (TBCT) NFs

Bi‐Cu_7_Te_4_ NFs (20 mg), EDCI (191.7 mg), and NHS (122 mg) were dissolved in DMF (20 mL) and activated for 1 h. (2‐aminoethyl) triphenylphosphonium bromide (38.6 mg in 1 mL DMF) was added, and the reaction continued for 24 h. The product was isolated by centrifugation (8000 rpm, 8 min), washed extensively with deionized water, and dried to obtain TBCT NFs.

### 4.3 Electrochemical Measurements

A CHI 660E electrochemical analyzer was employed to record the thermoelectric currents generated by TBCT NFs under various thermal gradients. A three‐electrode configuration was adopted, consisting of a Pt plate as the counter electrode and an Ag/AgCl electrode as the reference, with the measurements conducted in 0.5 M Na_2_SO_4_ aqueous solution. For the working electrode fabrication, 20 µL of the ethanolic dispersion was dropcast onto a fluorine‐doped tin oxide (FTO) glass substrate. The dispersion itself was prepared by homogeneously mixing 10 mg of TBCT NFs into 2 mL of ethanol containing 1 mM Nafion.

### 4.4 Intracellular Grouping Configuration

Briefly, 4T1 cells were treated with (I) Control, (II) NIR, (III) NIR+ICE, (IV) TBCT NFs, (V) TBCT NFs + NIR, and (VI) TBCT NFs + NIR +ICE. The content of TBCT NFs in groups (IV), (V), and (VI) was 20 µg mL^−1^. Groups (II) and (V) were irradiated with an 808 nm laser (1.0 W cm^−2^) for 2 min. Groups (III) and (VI) used an ice pack as a cold source in a water bath to control the temperature to drop to 15°C quickly. While Group (VI) was under 808 nm laser (1.0 W cm^−2^) irradiation for 2 min and cooled by an ice bath (1 min) for five cycles. Subsequent treatments were conducted with the same experimental conditions as mentioned above.

### 4.5 Animals and Tumor Model

Pathogen‐free female BALB/c mice, aged 4–6 weeks, were obtained from Liaoning Changsheng Biotechnology Co., Ltd. For tumor implantation, each mouse was subcutaneously inoculated into the right axillary region with 100 µL of Matrigel suspension containing 4 × 10^6^ 4T1 mammary carcinoma cells. Once the tumors reached an average volume of about 100 mm^3^, the animals were randomly assigned to six experimental cohorts (n = 5 per group): (1) Control; (2) NIR irradiation alone (808 nm, 1.0 W cm^−2^); (3) cyclic thermal intervention, defined as icepack cooling applied during NIR irradiation (1.0 W cm^−2^) at 12, 24, and 48h intervals (five coolingirradiation cycles per session); (4) TBCT NFs alone (15 mg kg^−1^); (5) TBCT NFs (15 mg kg^−1^) combined with NIR laser (808 nm, 1.0 W cm^−2^); and (6) TBCT NFs (15 mg kg^−1^) plus cyclic thermal intervention (NIR irradiation plus icepack cooling at the same time points, 5 cycles per session). Over the entire experimental period, tumor dimensions and body weights were measured and documented daily. Tumor volume was estimated using the standard formula: volume = (length × width^2^)/2. After 15 days of treatment, all animals were humanely sacrificed. Major organs (heart, liver, spleen, lungs, and kidneys) as well as tumor tissues were collected for further histological and biochemical analyses. All procedures were carried out in strict accordance with the World Health Organization's International Guiding Principles for Biomedical Research Involving Animals, and the experimental protocol was reviewed and approved by the Animal Care and Use Committee of Harbin Medical University (Approval No. IRB3107724).

### 4.6 Statistical Analysis

Statistical analyses were performed using GraphPad Prism 8.0 software. Results are presented as mean ± SD. Mean values were compared using Student's t‐test. Non‐significant differences were denoted as ns, and significance levels were indicated by asterisks (*p < 0.05, **p < 0.01, ***p < 0.001, ****p < 0.0001).

## Author Contributions

**Haoyang Du** and **Chao Xu** performed the experiments, collected the data, analyzed the results, and wrote the manuscript draft. **Peng Liu**, **Wei Gao**, **Tao Ye**, and **Xinyuan Yu** assisted with conducting experiments and data collection. **Ying Shi** and **Jiaxin Liu** provided the necessary experimental resources. **Tiancong Zhao** assisted in the experimental design. **Bin Sun**, **Piaoping Yang**, and **Manjie Zhang** conceived the study, supervised the entire project, provided funding, and reviewed and edited the manuscript.

## Conflicts of Interest

The authors declare no conflicts of interest.

## Supporting information




**Supporting File**: advs78230‐sup‐0001‐SuppMat.docx.

## Data Availability

The data that support the findings of this study are available from the corresponding author upon reasonable request.
