# Supplementary material for: Targeted Perturbation of Mitochondrial Energy Metabolism via Thermoelectric Nanoflowers for Enhanced Cuproptosis Echoed With Immunotherapy
Source: Adv Sci (Weinh). 2026 Oct 14:e78230. Online ahead of print. doi: 10.1002/advs.78230 (PMC13667398; doi:10.1002/advs.78230)
Supplement: Supplementary file 1 [file ADVS-9999-e78230-s001.docx]

**Targeted perturbation of mitochondrial energy metabolism via thermoelectric nanoflowers for enhanced** **cuproptosis echoed with** **immunotherapy**

*Haoyang Du^#^, Chao Xu^#^, Hao Wang, Bo Peng, Peng Liu, Wei Gao, Tao Ye, Xinyuan Yu, Ying Shi, Jiaxin Liu, Tiancong Zhao, Bin Sun^*^, Piaoping Yang^*^, Manjie Zhang^*^*

**Experimental Section：**

**Materials**

Polyacrylic acid (PAA, *M*_w_ ≈ 1800) were purchased from Sigma (USA). Isopropyl alcohol (IPA), aqueous and ammonia solution were bought from Sinopharm Chemical Reagent Beijing Co, Ltd. Copper nitrate trihydrate (Cu(NO_3_)_2_·3H_2_O) were purchased from Macklin Inc. Bismuth trinitrate (Bi(NO_3_)_3_), 5,5'-dithiobis-(2-nitrobenzoic acid) (DTNB), 3,3',5,5'-Tetramethylbenzidine (TMB) were obtained from Aladdin. Potassium tellurite (K₂TeO₃) was bought from Energy Chemical. ATP7B, FDX1, Caspase-3, LIAS, BCL-2 and DLAT were obtained from Bioss Biotechnology Co, Ltd. 2-(4-Amidinophenyl)-6-indolecarbamidine dihydrochloride (DAPI), ATP assay kit, JC-1 assay kit, ROS assay kit, and Calcein/PI cell assay kit were obtained from Beyotime Biotechnology Co, Ltd. anti-CD45-APC-Cy7, anti-CD3-FITC, anti-CD8a-APC, anti-CD4-PE/Cy7, anti-CD11b-FITC, anti-Gr-1-PE, anti-F4/80-APC/Cy7, anti-CD206-PE-Cy7, anti-CD86-APC, anti-CD11c-FITC, anti-CD80-PE-Cy7, anti-MHC-II-PE, anti-CD44-PE, and anti-CD62L-APC-CY7 were obtained from Biolegend Biotechnology Inc. (Beijing, China). Deionized (DI) water (18.2 MΩ.cm) used in all experiments was prepared by Milli-Q system (Millipore, USA).

**Characterization**

Transmission electron microscopy (TEM) was performed on a JEOL-2100F transmission electron microscope under 200 kV accelerating voltage. Scanning electron microscopy (SEM) image and the energy dispersive X-ray (EDX) spectrum were recorded on an XL30 ESEM-FEG field-emission scanning electron microscope (FEI Co.). X-ray diffraction pattern (XRD) was obtained on a Rigaku Ultima IV diffractometer (Rigaku, Japan) with CuKα radiation (l = 0.15405 nm). The X-ray photoelectron spectrum (XPS) was carried out with an ECSALAB 250 using nonmonochromatized Al Kα radiation. Fourier transform infrared spectroscopy (FTIR) was obtained on a Magna 560 FTIR spectrometer (Nicolet, USA). Ultraviolet-visible (UV-vis) absorption spectroscopy was obtained on a UH5300 spectro-photometer (Hitachi, Japan). UV-vis-NIR absorption spectra were measured with a Cary 500 UV-vis spectrophotometer (Varian, USA). Zeta potential of the nanocomposites was determined using a dynamic light scattering (ZS90, Malvern, UK). Confocal laser scanning microscopy (CLSM) mages were acquired using a Leica TCS SP8 microscope. A continuous-wave (CW) diode laser (LSR808H) with wavelength of 808 nm was used for the laser irradiation experiment. Electron spin resonance (ESR) spectra were acquired on a Bruker EMXplus EPR spectrometer. In vivo fluorescence imaging was conducted on an IVIS Lumina imaging system (PerkinElmer, America).

**Photothermal Effect Measurement**

An aqueous solution of TBCT NFs at concentrations of 0, 25, 50, 100, and 150 μg/mL was subjected to 808 nm laser irradiation (1 W/cm^2^) for 5 min, with temperature variations measured every 30 seconds using an infrared thermal imaging camera. Additionally, a 50 μg/mL concentration of TBCT NFs solutions was exposed to an 808 nm laser with power densities of 0.5, 1.0, 1.5, and 2.0 W/cm^2^ for 5 min, and temperature variations were recorded. To examine the photothermal stability of the TBCT NFs in greater detail, the TBCT NFs aqueous solution was subjected to four heating-cooling cycles using an 808 nm laser at 1 W/cm^2^.The photothermal conversion efficiency (*η*) was computed using Equation (1):

$\eta=\frac{hS(T_{\max}-T_{surr})-Q_{dis}}{I(1-10^{-A_{808}})}$ (1)

*h* is the heat transfer coefficient, *S* is the surface area of the container, *T*_max_ is the equilibrium temperature, *T*_Sur_ is the surrounding ambient temperature, *Q*_dis_ represents the heat absorbed by the container, *I* is the laser power (1 W/cm^2^), and *A*_808_ is the absorbance of TBCT NFs at 808 nm. The value of *hS* is derived from the following equation (2):

$\tau_{\text{s}}=\frac{m_{\text{D}}C_{\text{D}}}{hS}$ (2)

τs is the thermal equilibrium time constant, *m*_D_ is the mass of the sample, and *C*_D_ is the specific heat capacity of water (4.2 J/g·K).

**Oxidation of NADH**

To investigate NADH oxidation during PTET, a mixture of TBCT NFs solution (50 μg mL^-1^) and NADH solution (50 μg mL^-1^) was irradiated using an 808 nm NIR laser (1.0 W cm^-2^) for 2 min (laser on), followed by natural cooling to ambient temperature (25°C) (laser off). This cycle was repeated three times. Absorbance changes at 340 nm (NADH) and 260 nm (NAD⁺) were monitored using a UV-vis spectrophotometer. Separately, to evaluate thermal cycling effects, 3 mL of the mixed TBCT NFs/NADH solution (identical concentrations) was alternately transferred between water baths maintained at 55°C and 15°C. During these experiments, the solution was wrapped in aluminum foil to prevent photochemical interference.

**Measurement of ·OH Generation**

The presence of ·OH was identified using 3,3',5,5'-tetramethylbenzidine (TMB). First, various concentrations of TBCT NFs (10, 20, 30, 40, and 50 μg/mL), H_2_O_2_ (0.5 mM), and TMB were thoroughly mixed and incubated for 15 min. The absorbance of supernatant was measured at 652 nm by UV spectrophotometer. To investigate the effects of reaction time and laser irradiation on ·OH production, a 20 μg/mL TBCT NFs solution was mixed with H_2_O_2_ (0.5 mM) and TMB and incubated for 0, 10, 20, 40, and 50 min, respectively. After centrifugation, the supernatant’s absorbance was measured at 652 nm. In addition, TBCT NFs (20 μg mL^-1^) were treated with various concentrations of H_2_O_2_ (62.5, 125, 250, 500, 1000 and 2500 μM) in 2 mL of PBS solution (pH 5.5) containing TMB (0.5 mM). Additionally, electron spin resonance (ESR) spectroscopy was employed to detect ·OH, using 5,5-dimethyl-1-pyrroline N-oxide (DMPO) as a trapping agent for ·OH. TBCT NFs (20 μg/mL), DMPO (1.0 M), H_2_O_2_ (0.5 mM), and PBS (1 mL) were mixed for 10 min, followed by ESR spectrum measurement.

**In Vitro Cytotoxicity Evaluation**

The therapeutic efficacy of TBCT NFs was evaluated using MTT assay. 4T1 cells were seeded in 96-well plates at a density of 4 × 10^3^ cells per well, ensuring an even distribution across wells. The experimental groups included: Control, NIR, NIR+ICE, TBCT NFs, TBCT NFs+NIR, and TBCT NFs+NIR+ICE were incubated overnight in a 37°C incubator with 5% CO_2_ to allow cell attachment. Experimental concentrations of TBCT NFs were set at 0, 2.5, 5, 10, and 30 μg/mL, with six replicates per concentration. For groups requiring laser irradiation, cells were treated with drugs for 6 h before being irradiated with 808 nm laser (0.8 W/cm^2^) for 5 min. Following irradiation, cells were incubated at 37°C for an additional 24 h. Next, 4T1 cells were washed once with PBS, and each well was supplemented with 100 μL of MTT solution (1 mg/mL), then incubated for 4 h. Each well’s supernatants were removed, and 150 μL of DMSO was added, then gently shaken for 10 minutes. Absorbance was measured at 490 nm by microplate reader to calculate cell viability. The biocompatibility assay for L929 fibroblasts was conducted under the same conditions.

**Detection of Cellular ROS Content**

4T1 cells were plated uniformly in 6-well plates containing fresh coverslips and incubated overnight at 37°C. Cells were then subjected to treatments with Control, NIR, NIR+ICE, TBCT NFs, TBCT NFs+NIR, and TBCT NFs+NIR+ICE for 6 h. The NIR+ICE and TBCT NFs+NIR+ICE groups were irradiated using an 808-nm NIR laser (1.0 W cm^-2^) for 2 min (laser on), followed by natural cooling to ambient temperature (25°C) (laser off). This cycle was repeated three times. The plates were treated with DCFH-DA solution and incubated in the dark for 30 min. Next, cells were immobilized for 30 min with a 4% paraformaldehyde solution, and the process was carried out under light-protected conditions. Finally, confocal laser scanning microscope was utilized to evaluate the cellular ROS.

**Detection of Mitochondrial Membrane Potential**

1 mL of JC-1 working solution along with 1 mL of cell culture medium was added to each dish. After mixing well, the dishes were incubated at 37°C with 5% CO_2_ for 30 min. Upon completion of staining, the medium was gently discarded, and cells were washed twice with pre-cooled PBS to remove unbound JC-1 probe, minimizing nonspecific fluorescence background. Finally, confocal laser scanning microscope was utilized to monitor the fluorescence intensity of JC-1 probe.

**Evaluation of Intracellular ATP Level**

Culture media containing different concentrations of TBCT NFs (0, 10, and 20 μg/mL) were added to each well. After 6 h of incubation, the TBCT NFs+NIR group was exposed to an 808 nm laser (1 W/cm^2^) irradiation for 5 min. The cells were subsequently incubated for an additional 12 h, after which ATP levels in each group were assessed using an ATP assay kit.

**Survival Study:** Healthy female BALB/c mice (4-6 weeks old) were acquired from Liaoning Changsheng Biotechnology Co. Each mouse was subcutaneously inoculated in the right axillary region with 100 μL Matrigel containing 4 × 10^6^ 4T1 cells. Once the tumor volume reached approximately 100 mm^3^, the mice were randomly assigned to six groups (n = 6 per group) and treated according to the same regimens described above. The mice were monitored daily for signs of morbidity, and survival curves were plotted using the Kaplan-Meier method. Statistical significance was set at p < 0.05. All analyses were performed using Origin 8.

**In vivo anti-tumor immunity:** To assess the in vivo immunity efficacy of TBCT NFs, a xenograft model was established through subcutaneous implantation of 4T1 cells (4 × 10^6^) into the right flank of female BALB/c mice (Liaoning Changsheng Biotechnology Co.). Thirty-six tumor-bearing mice were randomly divided into six groups (n = 6 per group) and treated under different conditions, containing (I) control (PBS); (II) NIR laser irradiation only (808 nm, 1.0 W/cm^2^); (III) Cycled thermal intervention: ice-pack cooling applied during 808 nm irradiation (1.0 W/cm^2^) at 12, 24, and 48 h (5 cycles per session); (IV) TBCT NFs only (15 mg kg^-1^); (V) TBCT NFs (15 mg kg^-1^) + NIR laser (808 nm irradiation, 1.0 W/cm^2^); (VI) TBCT NFs (15 mg kg^-1^) + cycled thermal intervention (808 nm irradiation + ice-pack cooling at 12, 24, and 48 h; 5 cycles/session). For groups (IV)-(VI), mice received tail vein injections of each treatment (15 mg/kg) every three days starting from Day 0. On day 15, the mice were euthanized. Single-cell suspensions were prepared from tumors and para-inguinal lymph nodes, and various immune cells were detected by flow cytometry.

**mRNA sequencing experimental method**：Briefly, 4T1 cells were treated under conditions: (I) Control and (VI) TBCT NFs + NIR + ICE for 12 h, consistent with the methods described above. Total RNA was extracted using the TRIzol reagent (Invitrogen, CA, USA) according to the manufacturer’s protocol. RNA purity and quantification were evaluated using the NanoDrop 2000 spectrophotometer (Thermo Scientific, USA). RNA integrity was assessed using the Agilent 2100 Bioanalyzer (Agilent Technologies, Santa Clara, CA, USA). The transcriptome sequencing and analysis were conducted by LC-Bio Technologies (Hangzhou) Co., Ltd.

**Figure S1.** Zeta potentials of BCT NFs and TBCT NFs.

**Figure S2.** C 1s core-level XPS spectra.

**Figure S3.** a) The average nanoparticle size and the corresponding PDI of TBCT NFs dispersed in RPMI 1640 supplemented with 10% fetal bovine serum (FBS) within 7 days. b) Dispersibility of TBCT NFs (100 μg/mL) in different physiological media. Data are displayed as mean ± S.D. (n = 3).

**Figure S4.** Accumulated releasing Cu from different conditions.

**Figure S5.** Electrical properties of TBCT NFs. a) Electrical resistivity ρ, b) electrical conductivity σ, and c) power factor for TBCT NFs.

**Figure S6.** a) Comparison of the photothermal conversion efficiency (η) of TBCT NFs with typical photothermal materials. b) Comparison of the thermal conductivity of TBCT NFs with typical inorganic thermoelectric materials at room temperature.

**Figure S7.** UV-vis diffuse reflectance spectrum of TBCT NFs. The bandgap estimated by the Tauc plot method is plotted in the inset.

**Figure S8.** a) UV-vis absorption spectra of oxidized TMB recorded under different concentrations and b) at different times, respectively.

**Figure S9.** UV-vis absorption spectra of oxidized MB recorded under a) different concentrations, b) at different times and c) at various conditions, respectively.

**Figure S10.** CLSM images of the colocalization of Cy 5.5-labeled TBCT NFs with mitochondria.

**Figure S11.** Intracellular ATP levels in 4T1 cells. a) ATP levels under different treatments. b) Time-dependent changes in ATP levels at 0, 0.5, 1, 2, 4, and 8 h after TBCT NFs+NIR+ICE treatment. Data are presented as mean ± SD (n = 6). Statistical significance was determined by one-way ANOVA with Tukey's post-hoc test.

**Figure S12.** Measurement of intracellular Cu concentration under different treatment conditions (n = 3). (Ⅰ) Control, (Ⅱ) NIR, (Ⅲ) NIR+ICE, (Ⅳ) TBCT NFs, (Ⅴ) TBCT NFs+NIR, (Ⅵ) TBCT NFs+NIR+ICE.

**Figure S13.** Intracellular Cu concentration in 4T1 cells. a) Time-dependent changes in Cu concentration at 0, 0.5, 1, 2, 4, and 8 h after TBCT NFs+NIR+ICE treatment. b) Comparison among different treatment groups. Data are presented as mean ± SD (n = 3).

**Figure S14.** Cell viabilities by TBCT NFs+NIR+ICE treatments in combination with the additions of tetrathiomolybdate (TTM).

**Figure S15.** ·O_2_^-^ level with different treatments.

**Figure S16.** Calcein AM/PI double staining for determining cell viability.

**Figure S17.** The expression levels of apoptosis-related proteins (Bax and Bcl-2) in 4T1 cells treated with various conditions examined by western blotting.

**Figure S18.** Biodistribution of a) Cu and b) Te in main tissues and tumors at indicated time points after intravenous injection. Data are presented as percentage of injected dose per gram of tissue (% ID/g). Values are shown as means ± SD (n = 3 biologically independent mice per group).

**Figure S19.** a) In vitro *T*_1_-MR images of TBCT NFs with different concentrations. b) Relaxation rate *r_1_ versus* Cu concentration of TBCT NFs.

**Figure S20.** Biocompatibility measurement of TBCT NFs by hemolysis assay.

**Figure S21.** a) CD8^+^ T cells in the spleen. b) CD4^+^ T cells in the spleen. c) Tcm in CD8^+^ T cells. d) Tcm in CD4^+^ T cells.

**Figure S22.** a) M2 TAMs in tumors determined by flow cytometry. b) Statistical analysis of maturation rates of M2 macrophages.

**Figure S23.** Normalized expression (FPKM) of key cuproptosis-related genes (Atp7b, Atp7a, Fdx1, and Lias) in 4T1 cells. Data are presented as mean ± SD (n = 3). Adjusted *p*-values were determined by t-test analysis.

**Figure S24.** Raw Western blot data.

**References**

[1] B. Sun, W. Gao, X. Yue, C. Zhang, H. Du, Y. Luo, J. Zhu, P. Yang, M. Zhang, *Biomaterials* **2025**, *315*, 122907.

[2] [Y. Dong](https://pubmed.ncbi.nlm.nih.gov/?term=Dong+Y&cauthor_id=37910608), [S. Dong](https://pubmed.ncbi.nlm.nih.gov/?term=Dong+S&cauthor_id=37910608), [C. Yu](https://pubmed.ncbi.nlm.nih.gov/?term=Yu+C&cauthor_id=37910608), [J. Liu](https://pubmed.ncbi.nlm.nih.gov/?term=Liu+J&cauthor_id=37910608), [S. Gai](https://pubmed.ncbi.nlm.nih.gov/?term=Gai+S&cauthor_id=37910608), [Y. Xie](https://pubmed.ncbi.nlm.nih.gov/?term=Xie+Y&cauthor_id=37910608), [Z. Zhao](https://pubmed.ncbi.nlm.nih.gov/?term=Zhao+Z&cauthor_id=37910608), [X. Qin](https://pubmed.ncbi.nlm.nih.gov/?term=Qin+X&cauthor_id=37910608), [L. Feng](https://pubmed.ncbi.nlm.nih.gov/?term=Feng+L&cauthor_id=37910608), [P. Yang](https://pubmed.ncbi.nlm.nih.gov/?term=Yang+P&cauthor_id=37910608), [Y. Zhao](https://pubmed.ncbi.nlm.nih.gov/?term=Zhao+Y&cauthor_id=37910608), *Sci*. *Adv*. **2023**, *9*, 9980.

[3] Y. Guo, S. Ding, C. Shang, C. Zhang, M. Li, Q. Zhang, L. Gu, B. Heng, S. Zhang, F. Mei, Y. Huang, X. Zhang, M. Xu, J. Jiang, S. Guo, X. Deng, L. Chen, *Adv*. *Mater*. **2024**, *36*, 8.

[4] Y. Shen, C. Li, R. Huang, R. Tian, Y. Ye, L. Pan, K. Koumoto, R. Zhang, C. Wan, Y. Wang, *Sci. Rep.* **2016**, *6*, 32501.

[5] H. Liu, X. Shi, F. Xu, L. Zhang, W. Zhang, L. Chen, Q. Li, C. Uher, T. Day, G. J. Snyder, *Nat. Mater*. **2012**, *11*, 422-425.

[6] Y. Kang, Q. Zhang, C. Fan, W. Hu, C. Chen, L. Zhang, F. Yu, Y. Tian, B. Xu, *J. Alloys Compd.* **2017**, *700*, 223-227.

[7] S. Kim, K. Lee, H. Mun, H. Kim, S. Hwang, J. Roh, D. Yang, W. Shin, X. Li, Y. Lee, G. Snyder, S. Kim, *Science* **2015**, *348*, 109-114.
